# Reduction‐Induced C─C Cleavage and Site‐Specific Hydrogenation of a Highly Strained Bilayer Spironanographene

**DOI:** 10.1002/anie.202510209

**Published:** 2025-06-23

**Authors:** Juan Lión‐Villar, Herdya S. Torchon, Yikun Zhu, Zheng Wei, Jesús M. Fernández‐García, Israel Fernández, Marina A. Petrukhina, Nazario Martín

**Affiliations:** ^1^ Departamento de Química Orgánica I Facultad de Ciencias Químicas Universidad Complutense Avda. Complutense s/n 28040 Madrid Spain; ^2^ Department of Chemistry University at Albany State University of New York 1400 Washington Avenue Albany NY 12222 USA; ^3^ IMDEA‐Nanociencia C/Faraday, 9, Campus de Cantoblanco Madrid 28049 Spain

**Keywords:** Bilayer spironanographene, Chemical reduction, DFT calculations, Hydrogenation, X‐ray crystallography

## Abstract

The chemical reduction of a bilayer spironanographene, **spiro‐NG** (C_137_H_120_), with Na and K metals in the presence of [2.2.2]cryptand to yield [Na^+^(2.2.2‐cryptand)](C_137_H_121_^−^) (**1**) and [K^+^(2.2.2‐cryptand)](C_137_H_121_^−^) (**2**), respectively, is reported. X‐ray crystallography reveals the formation of a new “naked” anion (**spiro‐NG_H_** ^−^), in which spirocyclic ring cleavage and subsequent hydrogenation have occurred. Density Functional Theory (DFT) calculations suggest that the generation of the radical anion of the parent nanographene (**spiro‐NG^•^** ^−^), upon electron acceptance from Na and K metals, induces the cleavage of the strained spirobifluorene core. The resulting spin density localizes on a particular carbon atom, previously attached to the spiranic sp^3^ carbon atom, facilitating a site‐specific hydrogenation to afford (**spiro‐NG_H_** ^−^). The electrostatic potential map of this anion reveals electron density concentrated at the five‐membered ring of the readily formed indenyl fragment, thus enhancing the aromaticity of the system. Furthermore, nuclear magnetic resonance (NMR) and UV–vis absorption spectroscopy experiments allowed to follow the in situ reduction and hydrogenation processes in detail.

## Graphical abstract

Single‐crystal X‐ray diffraction analysis revealed a reduction‐induced ring cleavage with subsequent hydrogenation of a bilayer spironanographene upon treatment with alkali metals. Density Functional Theory (DFT) calculations suggested localized spin density on the C‐atom at the central spirocycle upon one‐fold reduction, facilitating a site‐specific hydrogenation. The pronounced gain in aromaticity of the system also favored the formation of the hydrogenated product.

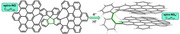

## Introduction

The discovery of graphene in 2004 by A. Geim and K. Novoselov marked the inception of the emergent 2D materials science.^[^ ^1^ ^]^ Over the past two decades,^[^ ^2^, ^3^ ^]^ these new materials have gained significant attention for diverse applications in next‐generation technologies, including optoelectronics,^[^ ^4^, ^5^, ^6^ ^]^ energy storage,^[^ ^7^, ^8^, ^9^ ^]^ sensors,^[^ ^10^, ^11^ ^]^ quantum computing,^[^ ^12^, ^13^ ^]^ and nanomedicine.^[^ ^14^, ^15^ ^]^ A further key milestone was achieved in 2010 with the observation of Van Hove singularities in twisted bilayer graphene,^[^ ^16^ ^]^ leading to the emergence of the so‐called moiré materials, thus starting the “twistronics” era.^[^ ^17^, ^18^, ^19^ ^]^ This area of research focuses on two or few layered materials able to exhibit superconductivity when two or more layers are twisted at a specific “magic angle”.^[^ ^20^, ^21^, ^22^, ^23^, ^24^ ^]^

Our group has similarly pioneered the development of molecular derivatives known as (chiral) bilayer nanographenes,^[^ ^25^ ^]^ which exhibit remarkable optoelectronic and chiroptical properties such as valence mixed states, excimer‐like behavior or high dissymmetry factors.^[^ ^26^ ^]^ In recent years, the extensive range of organic reactions available in synthetic chemistry has enabled the preparation of nanographenes with different sizes and shapes,^[^ ^27^, ^28^, ^29^, ^30^, ^31^ ^]^ including van der Waals molecular nanographene bilayers,^[^ ^32^, ^33^, ^34^, ^35^ ^]^ bilayers composed of fused radicals,^[^ ^36^, ^37^ ^]^ and covalently linked bilayers.^[^ ^38^, ^39^, ^40^, ^41^, ^42^, ^43^, ^44^ ^]^

**Figure 1:**
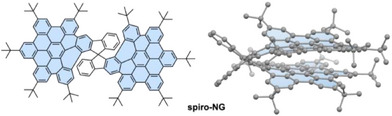
Chemical structure of **spiro‐NG** (left), and bilayer conformation stabilized by π–π interactions between both layers (right).^[^ ^45^ ^]^

In this context, we have very recently described the preparation of **spiro‐NG** (Figure 1).^[^ ^45^ ^]^ Intriguingly, the π‐π interactions between the two layers of hexa‐*peri*‐hexa‐benzocoronene (HBC) are sufficiently strong to modify the structure of this molecule from the usual orthogonal geometry featured by spirocompounds, to a favored bilayer structure (Figure 1, right). Similar to the related helical bilayer nanographenes,^[^ ^26^ ^]^ the mixed valence effect observed in this spironanographene has significant electronic implications.

Therefore, considering the structural singularity of this bilayer carbon nanostructure (**spiro‐NG**), we decided to explore its chemical reduction behavior with alkali metals, for the potential development of new energy storage systems.^[^ ^46^, ^47^, ^48^, ^49^ ^]^ In this context, molecular nanographenes have proven to be promising candidates, as they undergo multiple redox processes in the presence of alkali metals.^[^ ^50^, ^51^, ^52^, ^53^ ^]^ Furthermore, bilayer nanographenes have been demonstrated to accept several electrons when reacting with K or Rb, leading to the stepwise formation of trianions. Additionally, a reduction‐induced site‐specific double hydrogenation took place, provoking dramatic changes in the (electronic) structure of the nanographene.^[^ ^54^ ^]^

Herein, we report the chemical reduction of **spiro‐NG** (Figure 1) in the presence of Na and K metals and [2.2.2]cryptand, used as a secondary ligand to facilitate product crystallization. The successful X‐ray diffraction characterization of the resulting crystals confirmed the formation of the products, [Na^+^(2.2.2‐cryptand)](C_137_H_121_^−^) (**1**) and [K^+^(2.2.2‐cryptand)](C_137_H_121_^−^) (**2**), where the breaking of a C─C bond of the central spirocycle with subsequent hydrogenation, led to the “naked” **spiro‐NG_H_** ^−^ monoanion. Furthermore, the mechanism of this unusual reduction‐induced C─C cleavage followed by site‐specific hydrogenation process has also been studied by computational Density Functional Theory (DFT) methods.

## Results and Discussion

**Scheme 1:**
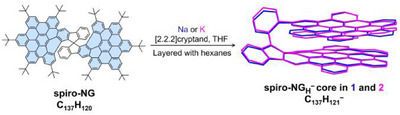
Chemical reduction of **spiro‐NG** with Na and K metals to afford the products **1** and **2**, cationic moieties and tert‐butyl groups are omitted for structure clarity.

Chemical reduction of **spiro‐NG** has been investigated with Na and K metals in anhydrous THF in the presence of [2.2.2]cryptand, at room temperature, under inert atmosphere. The reaction mixtures changed the color from the initial bright‐yellow (neutral ligand) through reddish‐brown, within 30–40 min, to afford vibrant green solutions which, upon slow diffusion of hexanes at 5 °C, produced the corresponding single crystals (Scheme 1).

**Figure 2:**
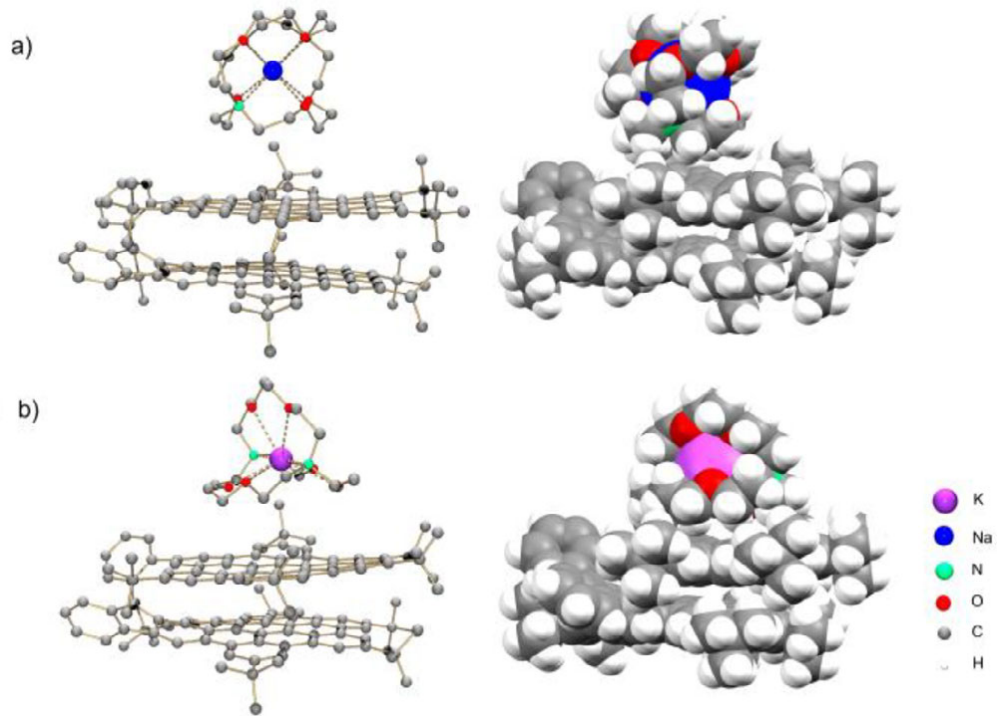
Crystal structures of a) **1** and b) **2**, ball‐and‐stick (H‐atoms are omitted for structure clarity) and space‐filling models. All M − O_crypt_ (2.459(4) − 2.548(4) Å for **1** and 2.771(17) − 2.874(18) Å for **2**) and M − N_crypt_ (2.684(4) − 2.703(4) Å for **1** and 3.039(2) − 3.060(3) Å for **2**) distances are close to the reported values.^[^ ^55^, ^56^, ^57^ ^]^

Their X‐ray diffraction analysis revealed the formation of solvent‐separated ion pairs (SSIPs) of **spiro‐NG_H_^−^** anion with one sodium counterion [Na^+^(2.2.2‐cryptand)](C_137_H_121_^−^) (**1**, crystallized with five interstitial THF molecules as **1**·5THF), or one potassium counterion [K^+^(2.2.2‐cryptand)](C_137_H_121_^−^) (**2**, crystallized with four interstitial THF molecules as **2**·4THF). In both cases, the Na^+^ or K^+^ ions are fully encapsulated by the [2.2.2]cryptand molecule and avoid direct metal‐π interactions with the monoanionic core, thus providing a “naked” **spiro‐NG_H_** ^−^ anion (Figure 2).

**Figure 3:**
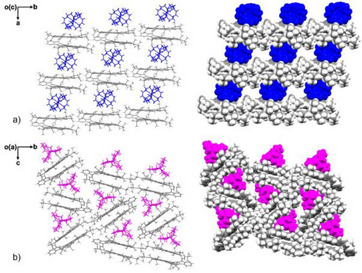
Solid‐state packing in a) **1** and b) **2**, capped‐stick and space‐filling models. [Na^+^(2.2.2‐cryptand)] and [K^+^(2.2.2‐cryptand)] moieties are shown in blue and pink, respectively.

Although no direct metal‐ion binding is observed in both crystal structures, multiple secondary C─H⋯π interactions (2.547(14) − 2.800(14) Å) between [Na^+^(2.2.2‐cryptand)] cationic moieties and the **spiro‐NG_H_^−^** anion contributed to the formation of the 1D stacks in the solid‐state structure of **1** (Figure 3a). In **2**, infinite zigzag 1D columns are also formed through C─H⋯π interactions (2.418(13) − 2.879(13) Å) between neighboring [K^+^(2.2.2‐cryptand)] moieties and the **spiro‐NG_H−_** anions (Figure 3b). Unlike the fully aligned stacked columns observed in **1**, the alternating building blocks in **2** adopt a staggered packing arrangement, which enables additional intercolumn C─H⋯π interactions. This staggered packing is commonly seen in bilayer frameworks to minimize steric hindrance.^[^ ^32^, ^33^, ^34^, ^35^ ^]^

**Figure 4:**
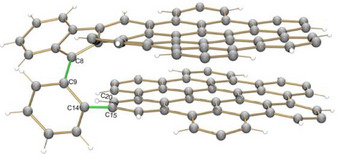
The core of **spiro‐NG_H_** ^−^ in **1** and **2**, ball‐and‐stick‐model. *Tert*‐butyl groups are omitted for structure clarity.

A careful crystallographic analysis of the anionic core reveals a major structural rearrangement of the spirocyclic framework in both crystalline products of **1** and **2**, in comparison with the neutral parent. In both structures, a C─C bond breakage (C8–C20, Figure 4) coupled with hydrogenation was observed in the spiro‐linkage of the two HBC layers. According to the structural analysis, a significant elongation of the C8─C9 and C14─C15 bonds (ranging over 1.470(3)–1.494(3) Å, highlighted in green) along with an averaged bond angle of 122.2° at C20 was observed in **1** and **2**, illustrating the ring cleavage and charge density localization at the spirocycle core. Both parameters also indicate the site‐specific hydrogenation of the C20 atom.

**Figure 5:**
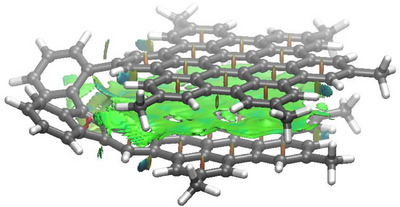
Contour plot of the reduced density gradient isosurface (density cutoff of 0.045 a.u.) computed for compound **1M‐H** ^−^ (*tert‐*Bu groups replaced by Me groups). The green surfaces indicate attractive noncovalent interactions.

Furthermore, it could also be noted that the addition of one electron to this spiro nanographene leads to an increase of nonplanarity of the HBC layers reflected by the continuous increase of dihedral angles between the peripheral six‐membered rings A/E (8.78°), B/E (9.55°), and C/E (10.70°) in **2** and resulting in the formation of a twisted **spiro‐NG_H_** ^−^ bilayer (See Table S2). Despite that, the two HBC units display high π‐surface overlap with multiple interplanar C─C contacts, resulting in significant π–π interactions with distances ranging over 3.34(2)–3.66(2) Å in **1** and 3.43(2)–3.64(2) in **2** (See Figure S10). These values are comparable to π‐stacks of unsubstituted neutral HBC (3.42 Å) and are only slightly larger than that of graphite (3.35 Å).^[^ ^58^ ^]^ The strong π–π interactions between the two layers were also visualized with the help of the non‐covalent interaction plot (NCIPlot) method, which clearly shows the occurrence of an extended noncovalent attractive interaction (green surface in Figure 5) between the aromatic rings of both HBC moieties.

**Figure 6:**
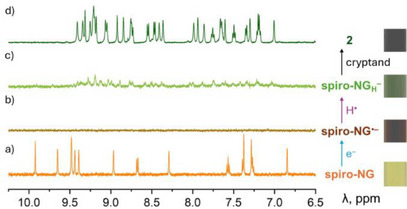
^1^H NMR spectra of a) spiro‐NG and b)–d) in situ generated anions of **spiro‐NG** under different conditions in THF‐*d*_8_, aromatic region. b) K/**spiro‐NG**, after 5 minutes; c) solution from b) sitting without K metal for 20 min; d) K/**spiro‐NG**/[2.2.2]cryptand, after 30 min.

The reduction of **spiro‐NG** with potassium metal, followed by hydrogenation, was monitored by ^1^H NMR spectroscopy (Figure 6). **Spiro‐NG** was dissolved in THF‐*d*₈ to afford a yellow solution (Figure 6a). Upon addition of potassium, the solution turned brown, and the NMR signals disappeared, which is consistent with the formation of the paramagnetic open‐shell species **spiro‐NG^•−^** (Figure 6b). Subsequent hydrogenation led to the reappearance of NMR signals, indicating the formation of the **spiro‐NG_H−_** anion. These signals reflect a loss of symmetry and the emergence of an additional aromatic proton (Figure 6c and d, in the absence and presence of [2.2.2]cryptand, respectively).

**Figure 7:**
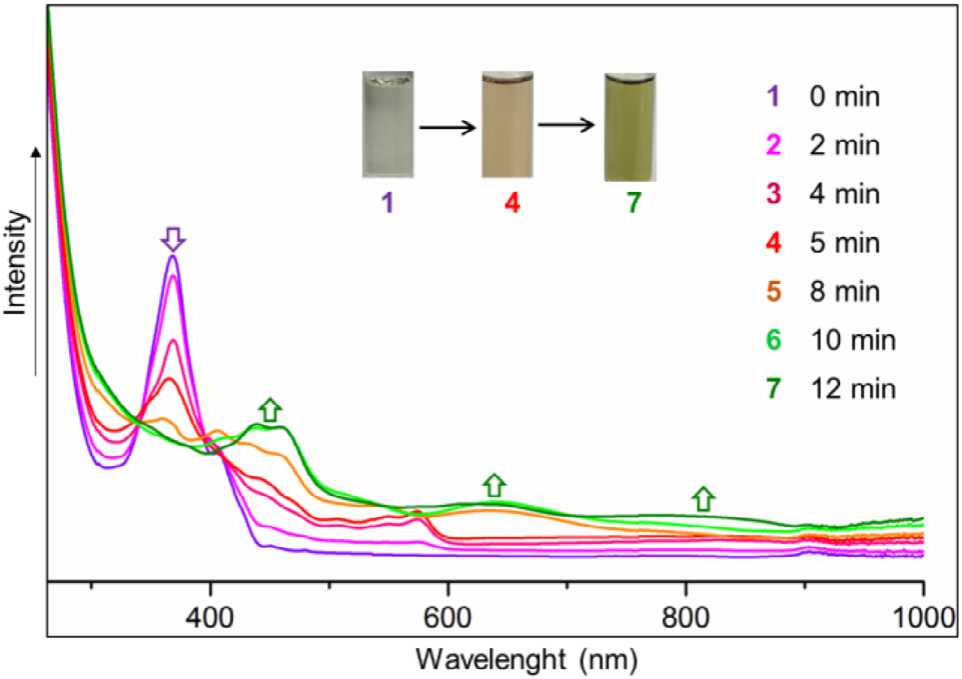
UV–vis spectra of in situ reduction of K/**spiro‐NG/**[2.2.2]cryptand in THF. UV–vis (THF, nm): *λ*_max_ 439, 459, and 648.

In addition, the reduction of **spiro‐NG** with potassium was also monitored by UV–vis spectroscopy (Figure 7). The initial absorption band of **spiro‐NG** at 368 nm completely disappears upon the addition of potassium, indicating the consumption of the neutral species. The resulting spectrum of the reduced compound displays new absorption bands at 439, 459, and 648, consistent with the formation of a highly conjugated anionic species.

Finally, we carried out DFT calculations at the dispersion‐corrected B3LYP‐D3/def2‐SVP level (see computational details in the Supporting Information) to gain more insight into the formation of the experimentally isolated anions. To this end, we focused on the model compound **1M**, where the bulky *tert*‐butyl groups in **spiro‐NG** were replaced by methyl groups.

**Figure 8:**
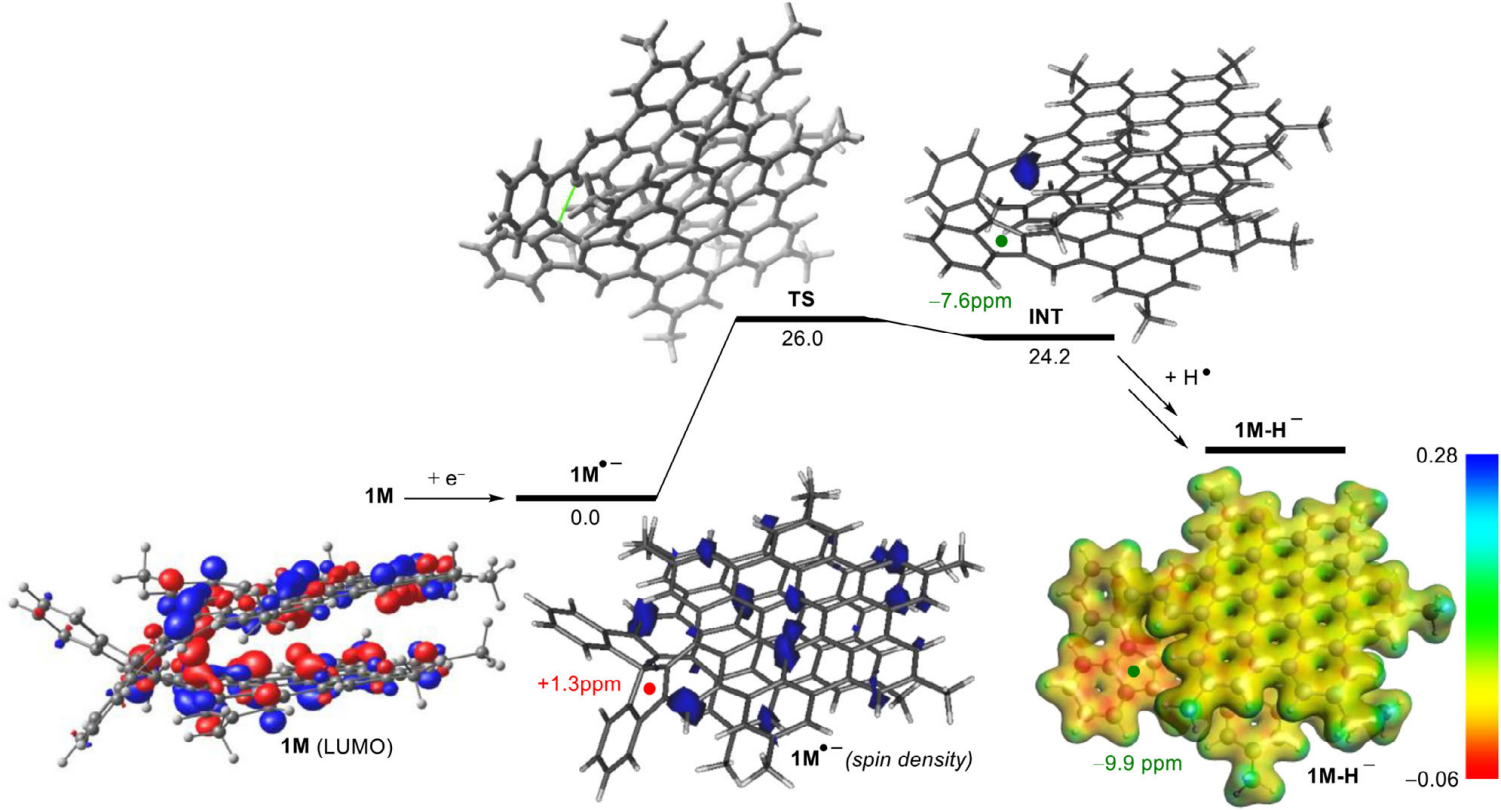
Computed key species for reduction‐induced ring cleavage and site‐specific hydrogenation of spironanographene **1M**. Relative free energies (Δ*G*, at 298 K) are given in kcal/mol. NICS values at the center of the five‐membered rings (denoted as a colored sphere) are given in ppm. All data have been computed at the B3LYP‐D3/def2‐SVP level.

Our calculations indicate that the LUMO of **1M** (the orbital accepting the electron from the alkali metal) is a π*‐molecular orbital delocalized in both HBC moieties (Figure 8). As a consequence, the reduction process produces the corresponding radical‐anion **1M^•^** ^−^, where the unpaired electron is fully delocalized in both HBC layers (as confirmed by the corresponding spin density plot in Figure 8). Once the reduction step has occurred, **1M^•−^** experiences a C─C bond‐rupture through the transition state (**TS**) with a feasible free activation barrier of ΔG^≠^ = 26.0 kcal mol^−1^. This saddle‐point is associated with the rupture of the C─C bond involving the spiranic carbon atom and produces the new radical‐intermediate **INT**. Interestingly, and in sharp contrast to the initial **1M^•−^** species, the computed spin‐density in this species indicates that the unpaired electron is exclusively localized at the carbon atom previously attached to the spiranic carbon atom. As a consequence, the subsequent hydrogenation reaction, which very likely occurs through a H abstraction from the medium (solvent or a trace of water), takes place exclusively at this position to produce the observed hydrogenated anion **spiro‐NG_H_^−^** (**1M‐H^−^** in the calculations).

Furthermore, the proposed mechanism for this rare reduction‐induced C─C cleavage followed by a site‐specific hydrogenation process involves that the negative charge in the final anion, as well as in the previous radical‐anion intermediate **INT**, should be concentrated at the readily formed indenyl fragment. Indeed, this is confirmed upon visualization of the computed electrostatic potential map of the model anion **1M‐H^v^** (Figure 8). As a result, the corresponding five‐membered ring becomes aromatic (i.e., resembling the cyclopentadienyl anion), as indicated by the computed negative Nuclear Independent Chemical Shift (NICS) values (−9.9 and −7.6 ppm, for **1M‐H^−^** and **INT**, respectively). This contrasts with the non‐aromatic nature of the initial spiranic radical‐anion **1M^•−^** (NICS = +1.3 ppm), which suggests that the gain in aromaticity in the process also contributes to the driving forces of the chemical transformation.

## Conclusions

In summary, we report the less‐known reduction‐induced C─C cleavage and site‐specific hydrogenation of a highly‐strained spiro bilayer nanographene with Na and K metals, yielding the respective salts [Na^+^(2.2.2‐cryptand)](C_137_H_121_^−^) (**1**) and [K^+^(2.2.2‐cryptand)](C_137_H_121_^−^) (**2**). X‐ray crystallography shows the formation of a new “naked” anion (**spiro‐NG_H_** ^–^), in which spirocyclic ring cleavage and subsequent hydrogenation have occurred. Interestingly, NMR experiments allowed us to follow the in situ reduction and hydrogenation processes. These results are underpinned by DFT calculations, which reveal that the reduction process localizes the resulting unpaired electron on a particular carbon atom of the previous spirobifluorene core, thus facilitating a site‐specific hydrogenation leading to **spiro‐NG_H_^−^**. Furthermore, the gain in aromaticity of the remaining five‐membered ring also contributes to the driving forces of the whole chemical transformation.

These experimental findings pave the way to a new type of redox reactivity in highly strained bilayer nanographenes, as well as to a better understanding of the structure‐reactivity trade‐off in these singular and less‐explored carbon nanoforms.

## Conflict of Interests

The authors declare no conflict of interest.

## Supporting information



Supporting Information

Supporting Information

## Data Availability

The data that support the findings of this study are available in the supplementary material of this article. For additional crystallographic data, see reference [59].
