# Supplementary material for: Reduction‐Induced C─C Cleavage and Site‐Specific Hydrogenation of a Highly Strained Bilayer Spironanographene
Source: Angew Chem Int Ed Engl. 2025 Jun 23;64(33):e202510209. doi: 10.1002/anie.202510209 (PMC12338388; doi:10.1002/anie.202510209)
Supplement: Supplementary file 1 — Supporting Information [file ANIE-64-e202510209-s001.docx]

Reduction-Induced C–C Cleavage and Site-Specific Hydrogenation of a Highly Strained Bilayer Spironanographene.

Juan Lión-Villar,^#[a]^ Herdya S. Torchon,^#[b]^ Yikun Zhu,^[b]^ Zheng Wei,^[b]^ Jesús M. Fernández-García,^[a]^ Israel Fernández,*^[a]^ Marina A. Petrukhina,*^[b]^ Nazario Martín*^[a,c]^

**Table of Contents**

[I. Materials and Methods 2](#_Toc200370192)

[II. UV-Vis Spectroscopic Investigation 4](#_Toc200370193)

[III. NMR Spectroscopic Investigation 5](#_Toc200370194)

[IV. EPR Spectroscopic Investigation 8](#_Toc200370195)

[V. Crystal Structure Solution and Refinement Details 9](#_Toc200370196)

[VI. Computational Details 16](#_Toc200370197)

[VII. References 35](#_Toc200370198)

# **I. Materials and Methods**

All manipulations were carried out using break-and-seal and glove-box techniques under an atmosphere of argon.^[1]^ Tetrahydrofuran (THF) and hexanes (Sigma Aldrich) were dried over Na/benzophenone and distilled prior to use. THF-*d*_8_ (Sigma Aldrich) was dried over NaK_2_ alloy and vacuum-transferred. Sodium (99.9 %), potassium (98 %) and [2.2.2]cryptand (99.0%) were purchased from Sigma Aldrich and used as received. C_137_H_120_ (**spiro-NG**) was prepared according to the previously reported procedure.^[2]^ The UV-Vis absorption spectra were recorded on a Shimadzu UV-2600i UV-Vis spectrophotometer. The ^1^H NMR spectra were recorded on a Bruker Ascend-500 spectrometer (500 MHz for ^1^H). Chemical shifts (*δ*) are reported in parts per million (ppm) and referenced to the resonances of the corresponding solvent used. The low-temperature NMR experiment was controlled by a Cryo Diffusion cryogenic tank probe, and liquid N_2_ was used as a cooling source. The EPR spectra were collected with an ADANI SPINSCAN X EPR spectrometer with the following parameters: center field = 335.00 mT, sweep width = 15 mT, sweep time = 60 s, modulation amplitude = 100 uT, attenuation = 20 dB, temperature = 30°C. The oxygen- and moisture sensitivity of the reduced products, **1** and **2**, along with the presence of multiple interstitial THF molecules, prevented obtaining elemental analysis data.

**[{Na^+^(2.2.2-cryptand)}(C_137_H_121_^–^)]·5THF (1·5THF)**

THF (1.0 mL) was added to a customized glass system containing excess Na metal (4.0 mg, 0.17 mmol), **spiro-NG** (3.0 mg, 0.002 mmol) and [2.2.2]cryptand (2.4 mg, 0.006 mmol). The reaction mixture was left to stir at 25 °C under argon for 40 minutes. The initial bright-yellow color (neutral ligand) changed to green after 20 minutes, and remained the same until the reaction was stopped. The mixture was then filtered, and the green filtrate was layered with 1.5 mL of anhydrous hexanes. The ampule was sealed under argon and stored at 5 °C. After 4 weeks, black plate-shaped crystals were deposited in the ampule. Yield: 4.8 mg, 43%.

**[{K^+^(2.2.2-cryptand)}(C_137_H_121_^–^)]·4THF (2·4THF)**

THF (1.2 mL) was added to a customized glass system containing excess K metal (5.0 mg, 0.13 mmol), **spiro-NG** (3.0 mg, 0.002 mmol) and [2.2.2]cryptand (3.0 mg, 0.008 mmol). The reaction mixture was left to stir at 25 °C under argon for 30 minutes. The initial bright-yellow color (neutral ligand) changed to green almost immediately, and remained the same until the reaction was stopped. The mixture was then filtered, and the green filtrate was layered with 2.0 mL of anhydrous hexanes. The ampule was sealed under argon and stored at 5 °C. After 4 weeks, black plate-shaped crystals were deposited in the ampule. Yield: 4.5 mg, 40%.

# **II. UV-Vis Spectroscopic Investigation**

**Sample preparation:** THF (3.0 mL) was added to a glass ampule (O.D. 12 mm) containing **spiro-NG** (0.4 mg, 0.0003 mmol), [2.2.2]cryptand (0.2 mg, 0.0005 mmol), and Na or K metals (1.0-2.0 mg) under inert atmosphere. The ampule was sealed with a Teflon stopper under argon. UV-Vis absorption spectra were monitored at different reaction times at room temperature.

**
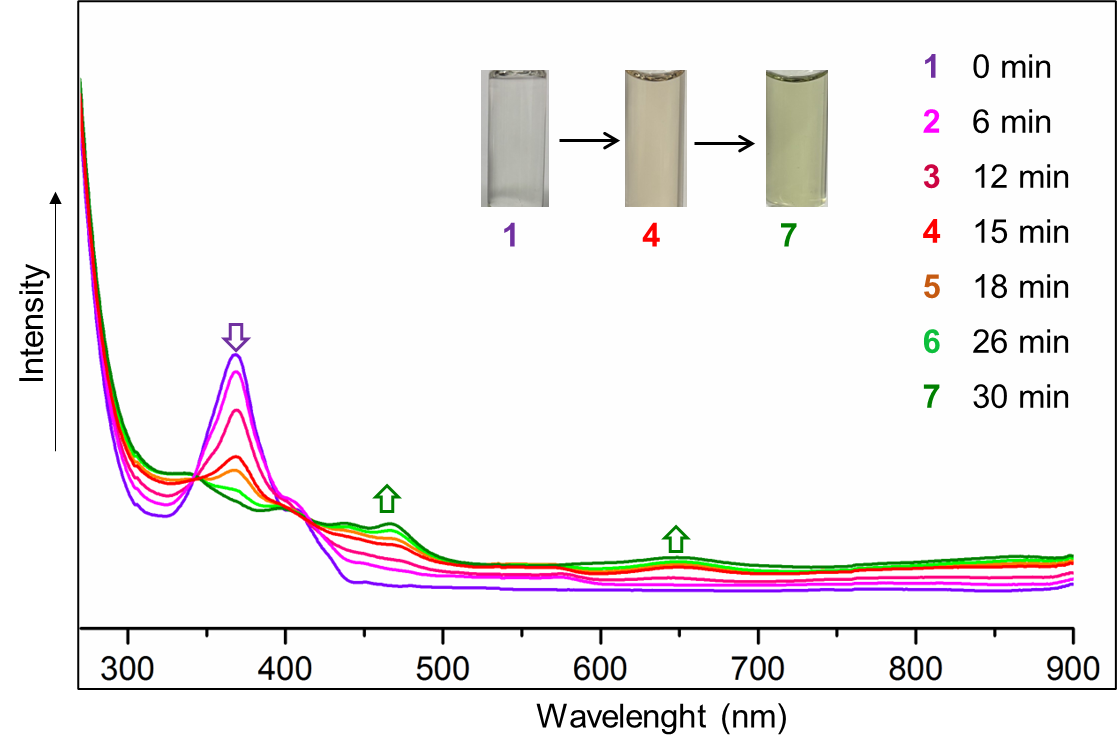
**

**Figure S1.** UV-Vis spectra of *in situ* reduction of Na/**spiro-NG/**[2.2.2]cryptand in THF. UV-Vis (THF, nm): *λ*_max_ 440, 465, 648.

# **III. NMR Spectroscopic Investigation**


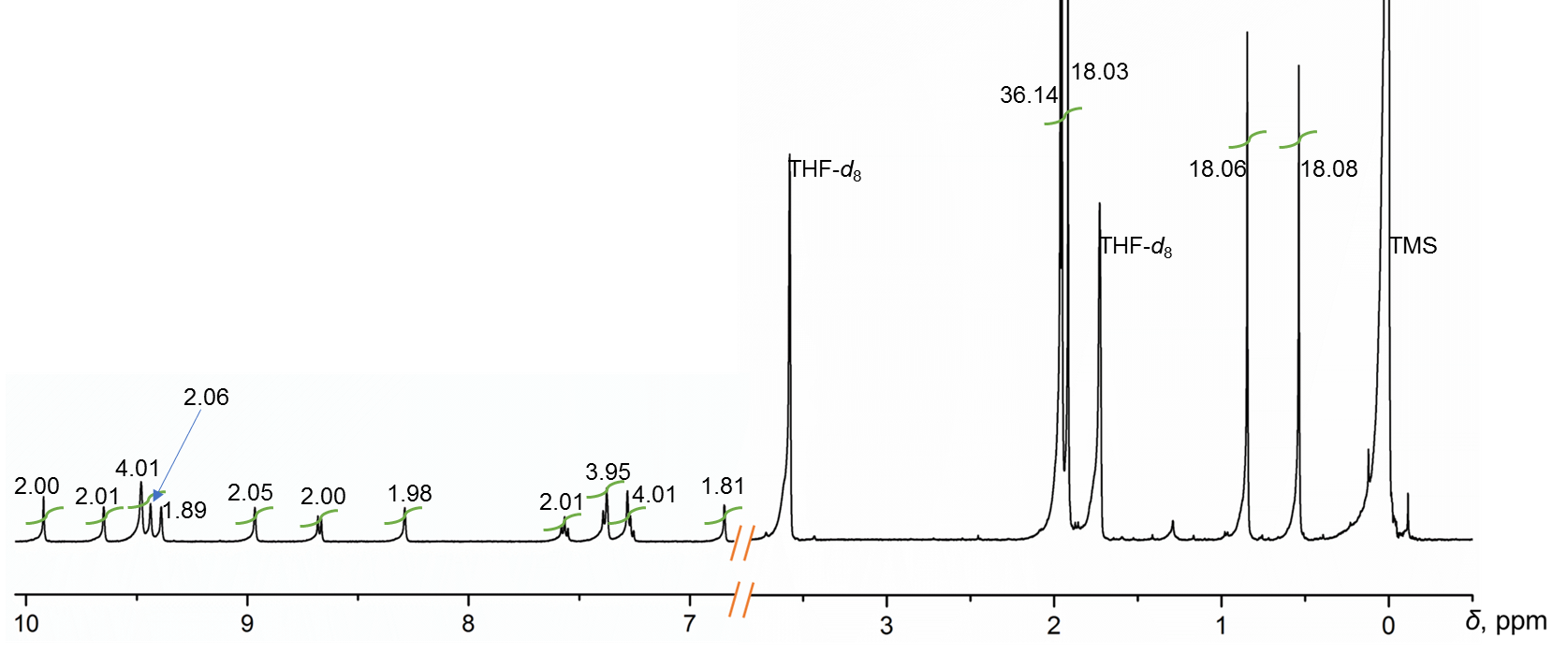


**Figure S2.** ^1^H NMR spectrum of **spiro-NG** (C_137_H_120_) in THF-*d*_8_ at 25℃, with integrations. ^1^H NMR (THF-*d*_8_, ppm, 25 ℃): 0.54 (18H), 0.85 (18H), 1.92 (18H), 1.97–1.95 (36H), 6.85 (2H), 7.26–7.28 (4H), 7.38–7.39 (4H), 7.55–7.58 (2H), 8.29 (2H), 8.67–8.68 (2H), 8.97 (2H), 9.39 (2H), 9.44 (2H), 9.48 (4H), 9.65 (2H), 9.92 (2H).

***Sample preparation:*** THF-*d*_8_ (0.60 mL) was added to an NMR tube containing excess K metal (2.0 mg, 0.051 mmol) and **spiro-NG** (1.0 mg, 0.001 mmol), with or without the presence of secondary ligand [2.2.2]cryptand (1.0 mg, 0.003 mmol). The tube was sealed under argon. ^1^H NMR spectrum of **spiro-NG** was collected immediately followed by the spectra of *in* *situ* generated products collected at 25℃.


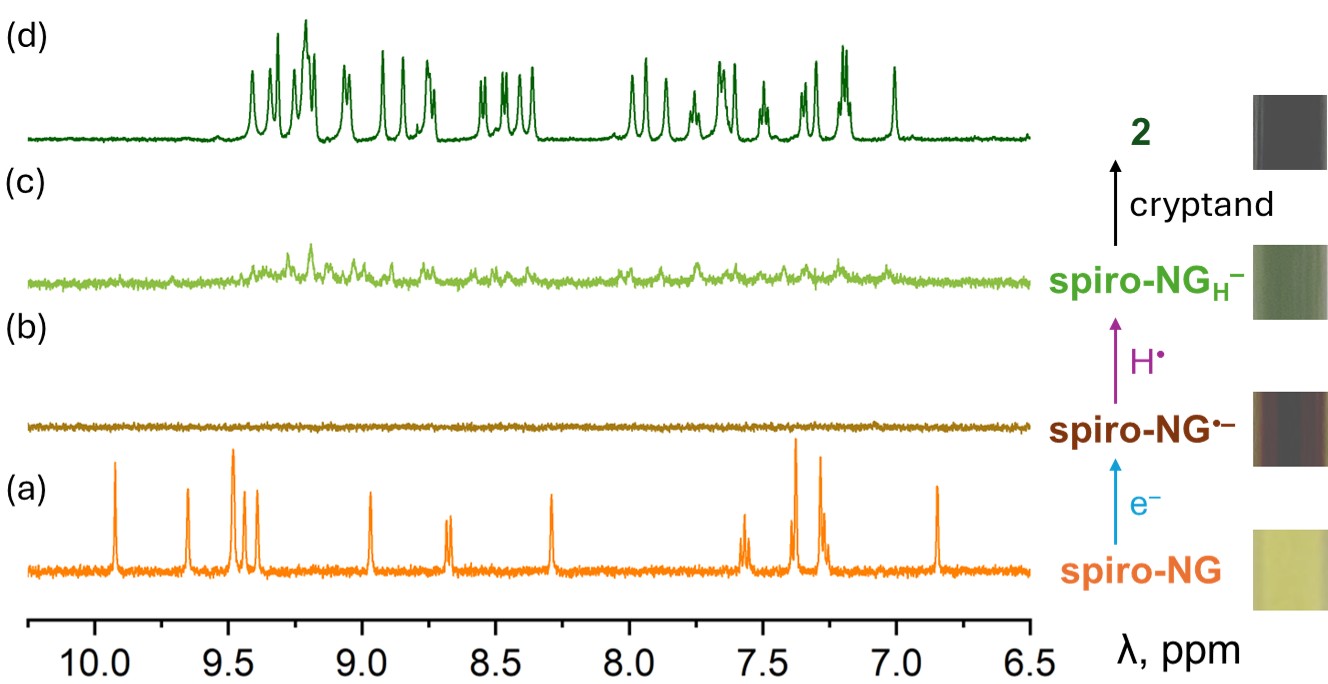


**Figure S3.** ^1^H NMR spectra of (a) **spiro-NG** and (b-d) *in* *situ* generated anions of **spiro-NG** under different conditions in THF-*d*_8_, aromatic region. (b) K/**spiro-NG**, after 5 minutes; (c) solution from (b) sitting without K metal for 20 minutes; (d) K/**spiro-NG**/[2.2.2]cryptand, after 30 minutes.


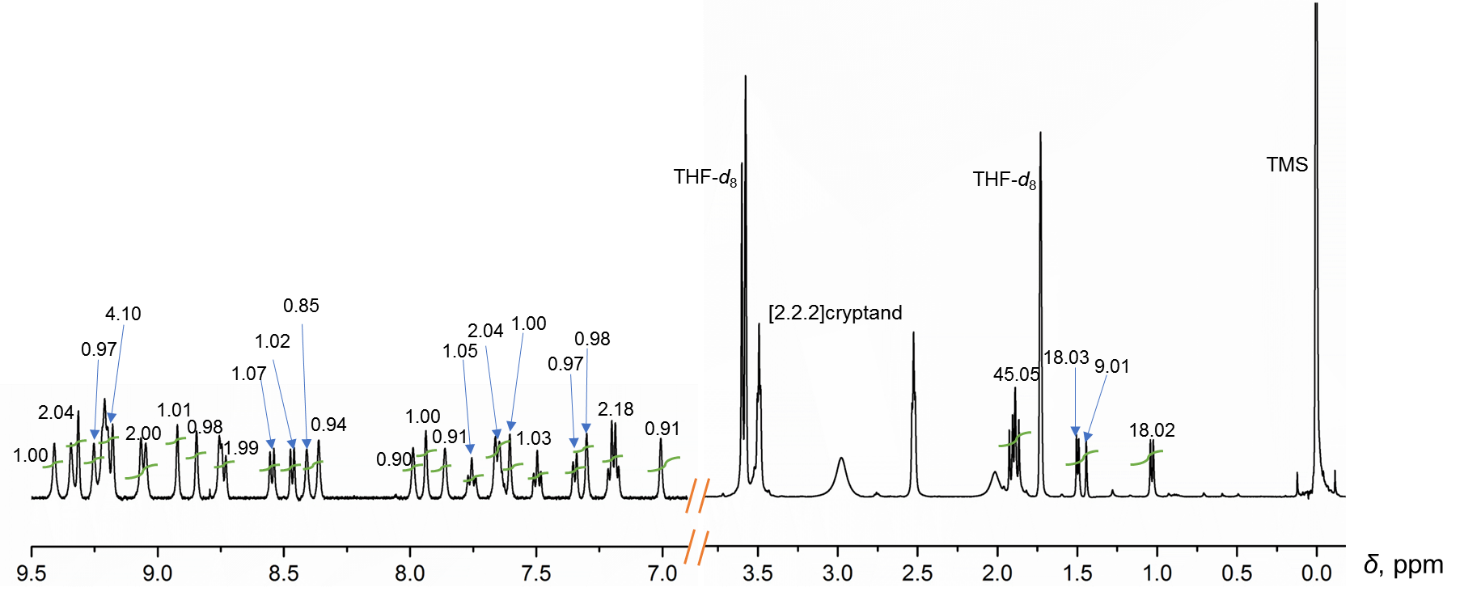


**Figure S4.** ^1^H NMR spectrum of *in* *situ* generated **spiro-NG_H_^–^** (C_137_H_121_^–^) in **2** in THF-*d*_8_ at –20℃, with integrations. ^1^H NMR (THF-*d*_8_, ppm, –20 ℃): 1.02–1.04 (18H), 1.44 (9H), 1.49–1.51 (18H), 1.87–1.93 (45H), 7.01 (1H), 7.17–7.22 (2H), 7.30 (1H), 7.34–7.35 (1H), 7.48–7.51 (1H), 7.60 (1H), 7.65–7.66 (2H), 7.74–7.77 (1H), 7.86 (1H), 7.94 (1H), 7.99 (1H), 8.36 (1H), 8.41 (1H), 8.46–8.47 (1H), 8.54–8.55 (1H), 8.73–8.76 (2H), 8.85 (1H), 8.92 (1H), 9.05–9.07 (2H), 9.18–9.21 (4H), 9.25 (1H), 9.31–9.34 (2H), 9.41 (1H).


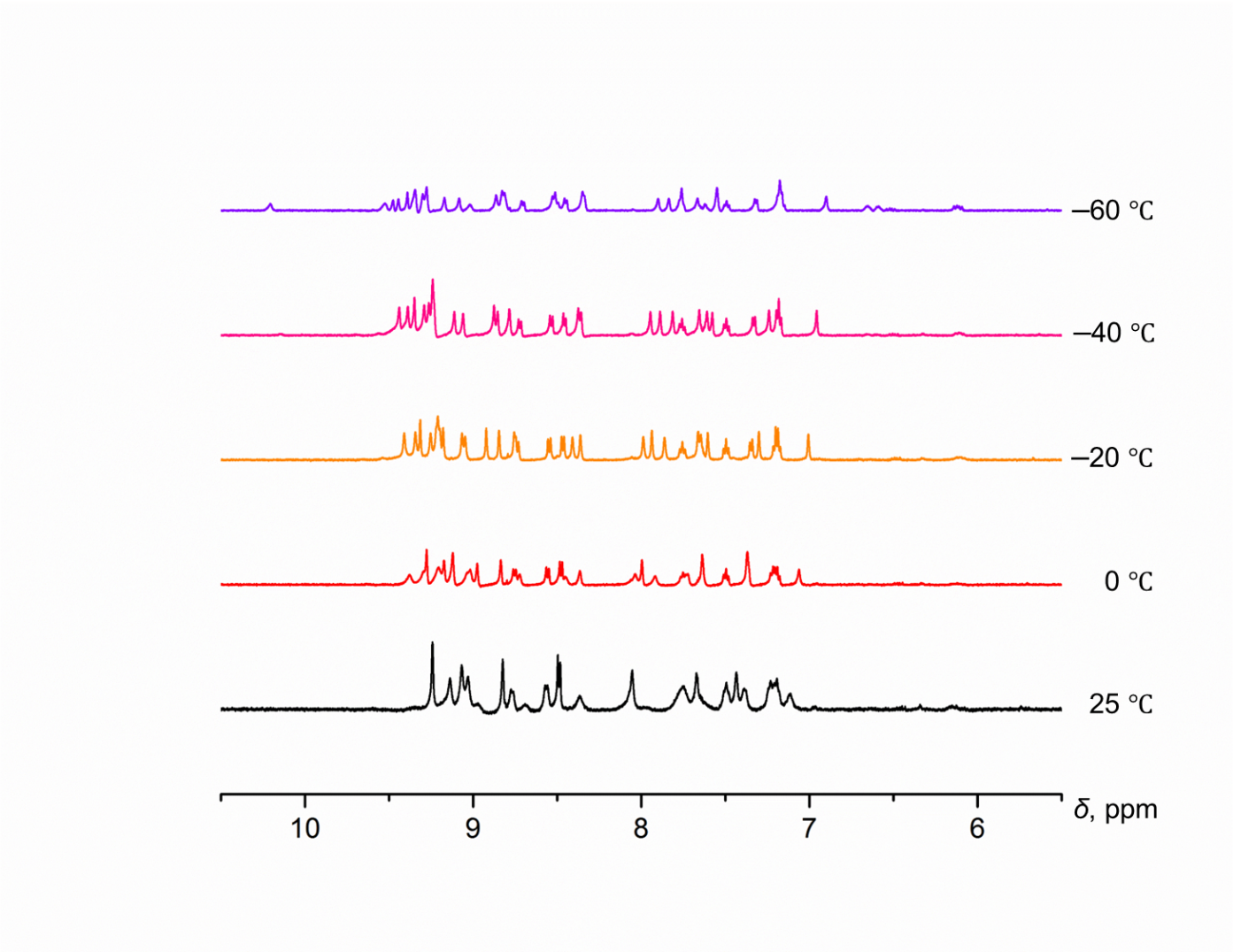


**Figure S5.** Variable-temperature ^1^H NMR spectra of *in* *situ* generated **spiro-NG_H_^–^** in **2** in THF-*d*_8_, aromatic region.

**Sample preparation:** Crystals of **2** (~2.0 mg) were washed with anhydrous hexanes, dried under vacuum, dissolved in anhydrous THF-*d*_8_ (0.70 mL), and transferred into an NMR ampule (O.D. 5 mm) under inert atmosphere. The NMR tube was sealed under argon, and ^1^H NMR spectra were collected at variable temperatures.


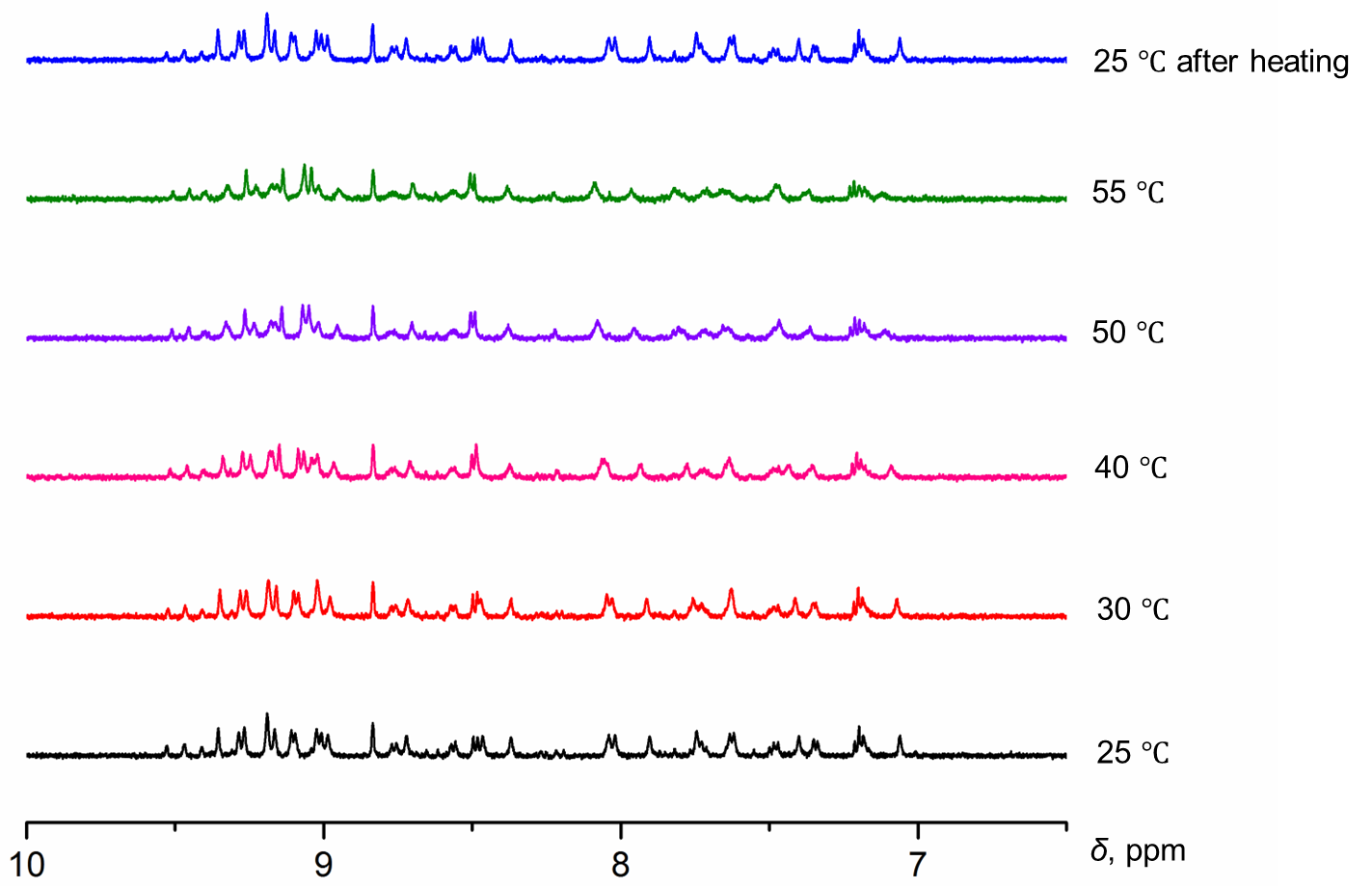


**Figure S6.** Variable-temperature ^1^H NMR spectra of crystals of **2** dissolved in THF-*d*_8_, aromatic region.

# **IV. EPR Spectroscopic Investigation**

**Sample preparation:** THF (0.8 mL) was added to a glass tube (O.D. 5 mm) containing excess K metal (2.0 mg, 0.051 mmol), **spiro-NG** (1.0 mg, 0.001 mmol), and [2.2.2]cryptand (1.2 mg, 0.003 mmol). The tube was sealed under argon, and EPR spectra were monitored at different reaction times at 30 °C.


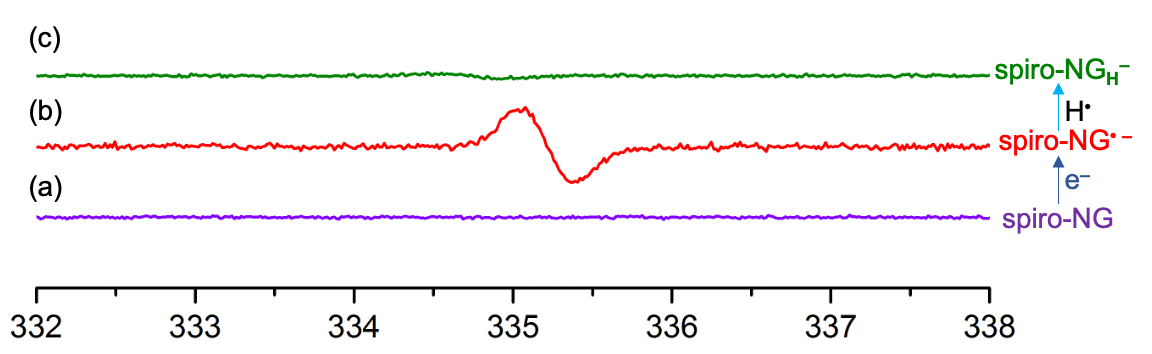


**Figure S7.** EPR spectra of *in situ* reduction of K/**spiro-NG**/[2.2.2]cryptand in THF.

# **V. Crystal Structure Solution and Refinement Details**

Data collections of **1**·5THF and **2**·4THF were performed at 100.00(10) K on a Rigaku XtaLAB Synergy-S X-ray diffractometer equipped with a HyPix-6000HE hybrid photon counting (HPC) detector and a microfocus Cu-K*α* radiation (*λ* = 1.54178 Å). Data collection strategy to ensure completeness and desired redundancy were determined using CrysAlisPro.^[3]^ Data processing was performed also using CrysAlisPro. Empirical absorption correction was applied using the SCALE3 ABSPACK scaling algorithm.^[4]^ The structures were solved by SHELXT (version 2018/2)^[5]^ and refined by full-matrix least-squares procedures using the Bruker SHELXTL (version 2019/3)^[6]^ software package through the OLEX2 graphical interface.^[7]^ All non-hydrogen atoms, including those in disordered parts, were refined anisotropically. Hydrogen atoms were included in idealized positions for structure factor calculations with *U*_iso_(H) = 1.2 *U*_eq_(C) and *U*_iso_(H) = 1.5 *U*_eq_(C) for methyl groups. In **1**·5THF, four *tert*-butyl groups and the whole [Na^+^(cryptand)] cationic unit was found to be disordered. In **2**·4THF, three *tert*-butyl groups were found to be disordered. The disordered parts were modeled with two orientations with their relative occupancies refined. The geometries of the disordered parts were restrained to be similar. They were also restrained to have the same *U*_ij_ components, with a standard uncertainty of 0.01 Å^2^. In each unit cell of **1**·5THF, ten THF solvent molecules were found to be severely disordered and removed by the Olex2’s solvent mask subroutine.^[7]^ The total void volume was 1,626.2 Å^3^, equivalent to 23.4 % of the unit cell’s total volume. In each unit cell of **2**·4THF, sixteen THF solvent molecules were found to be severely disordered and removed by the Olex2’s solvent mask subroutine.^[7]^ The total void volume was 3321.4 Å^3^, equivalent to 23.8 % of the unit cell’s total volume. Further crystal and data collection details are listed in Table S1. The ORTEP drawings, solid-state packings and additional structural figures, along with the key C–C bond distances and angles, are shown below.

**Table S1.** Crystallographic data of **1**·5THF and **2**·4THF.

| **Compound** | **1**·5THF | **2**·4THF |
| --- | --- | --- |
| Empirical formula | C_175_H_197_NaN_2_O_11_ | C_171_H_189_KN_2_O_10_ |
| Formula weight | 2527.32 | 2471.33 |
| Temperature (K) | 100.01(10) | 100.00(10) |
| Wavelength (Ǻ) | 1.54184 | 1.54184 |
| Crystal system | Triclinic | Monoclinic |
| Space group | *P*–1 | *P*2_1_*/c* |
| *a* (Å) | 15.24049(18) | 17.44165(18) |
| *b* (Å) | 17.2544(2) | 32.1737(4) |
| *c* (Å) | 28.8150(5) | 25.8287(3) |
| *α* (°) | 105.7418(13) | 90.00 |
| *β* (°) | 93.5343(12) | 105.4958(12) |
| *γ* (°) | 105.4929(11) | 90.00 |
| *V* (Å^3^) | 6954.30(18) | 13967.3(3) |
| *Z* | 2 | 4 |
| *ρ*_calcd_ (g·cm^-3^) | 1.207 | 1.175 |
| *μ* (mm^-1^) | 0.595 | 0.810 |
| *F*(000) | 2720 | 5312 |
| Crystal size (mm) | 0.09×0.13×0.16 | 0.09×0.12×0.22 |
| *θ* range for data collection (°) | 2.785–79.283 | 2.629–80.693 |
| Reflections collected | 83856 | 161898 |
| Independent reflections | 28281  [*R*_int_ = 0.0409] | 29247  [*R*_int_ = 0.0412] |
| Transmission factors (min/max) | 0.77964/1.00000 | 0.60948/1.00000 |
| Data/restraints/params. | 28281/1215/1901 | 29247/250/1608 |
| *R*1,^a^ *wR*2^b^ *(I* > 2*σ*(*I*)) | 0.1124, 0.3114 | 0.0688, 0.1973 |
| *R*1,^a^ *wR*2^b^ (all data) | 0.1268, 0.3239 | 0.0815, 0.2071 |
| Quality-of-fit^c^ | 1.026 | 1.053 |

*R*_int_ = Σ|*F*_o_^2^-<*F*_o_^2^>|/Σ|*F*_o_^2^|.

^a^*R*1 = Σ||*F*_o_|-|*F*_c_||/Σ|*F*_o_|. ^b^*wR*2 = [Σ[*w*(*F*_o_^2^-*F*_c_^2^)^2^]/Σ[*w*(*F*_o_^2^)^2^]].

^c^Quality-of-fit = [Σ[*w*(*F*_o_^2^-*F*_c_^2^)^2^]/(*N*_obs_-*N*_params_)]^½^, based on all data.


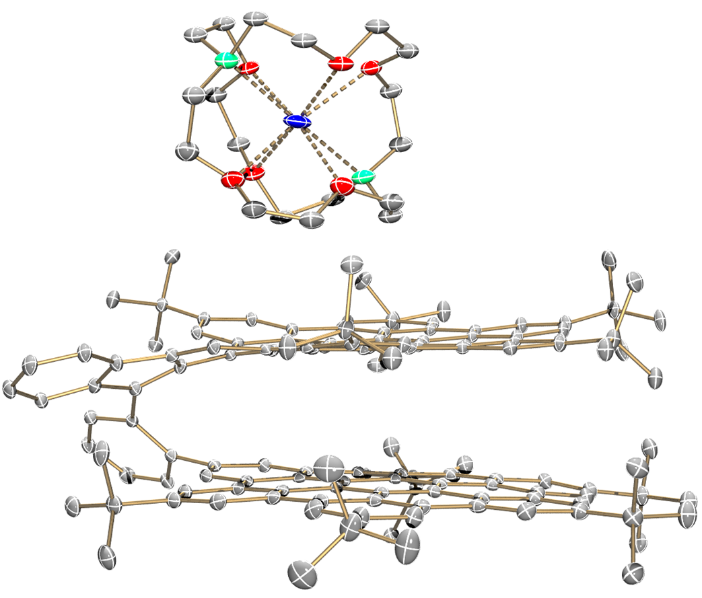


**Figure S8.** ORTEP drawing of the asymmetric unit of **1**, drawn with thermal ellipsoids at the 25% probability level. Color scheme used: C gray, O red, Na blue, N spring green.


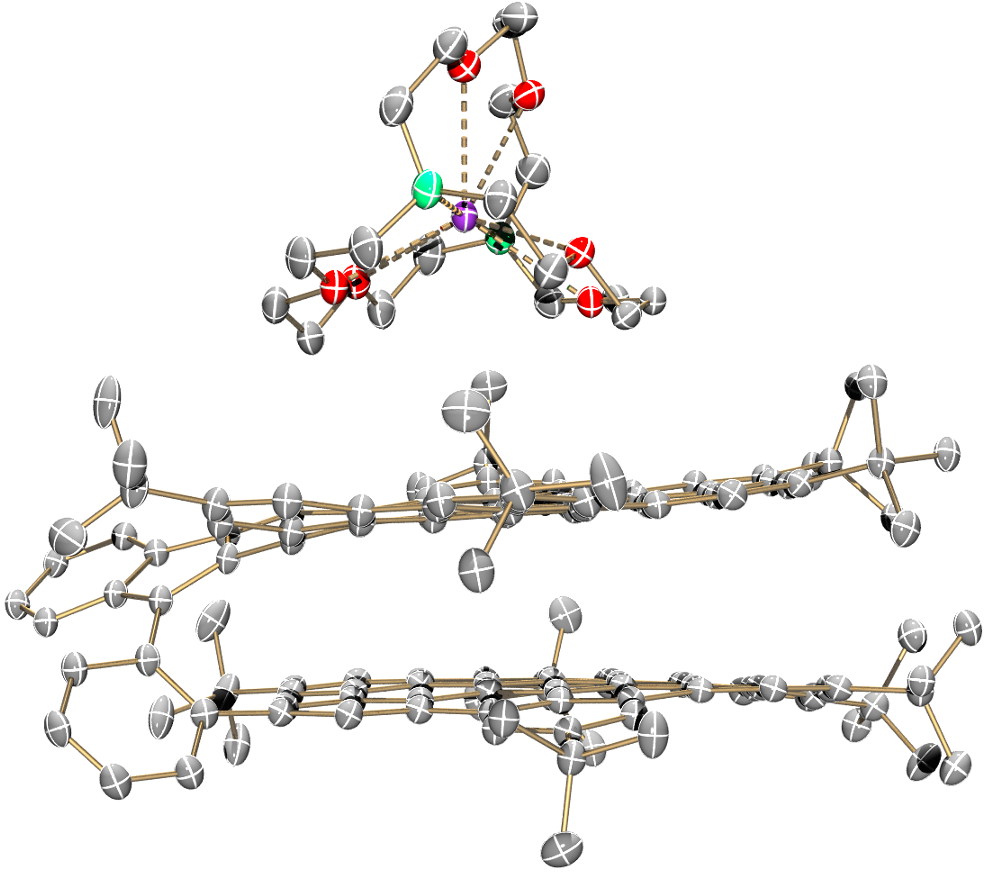


**Figure S9.** ORTEP drawing of the asymmetric unit of **2**, drawn with thermal ellipsoids at the 25% probability level. Color scheme used: C gray, O red, K dark orchid, N spring green.


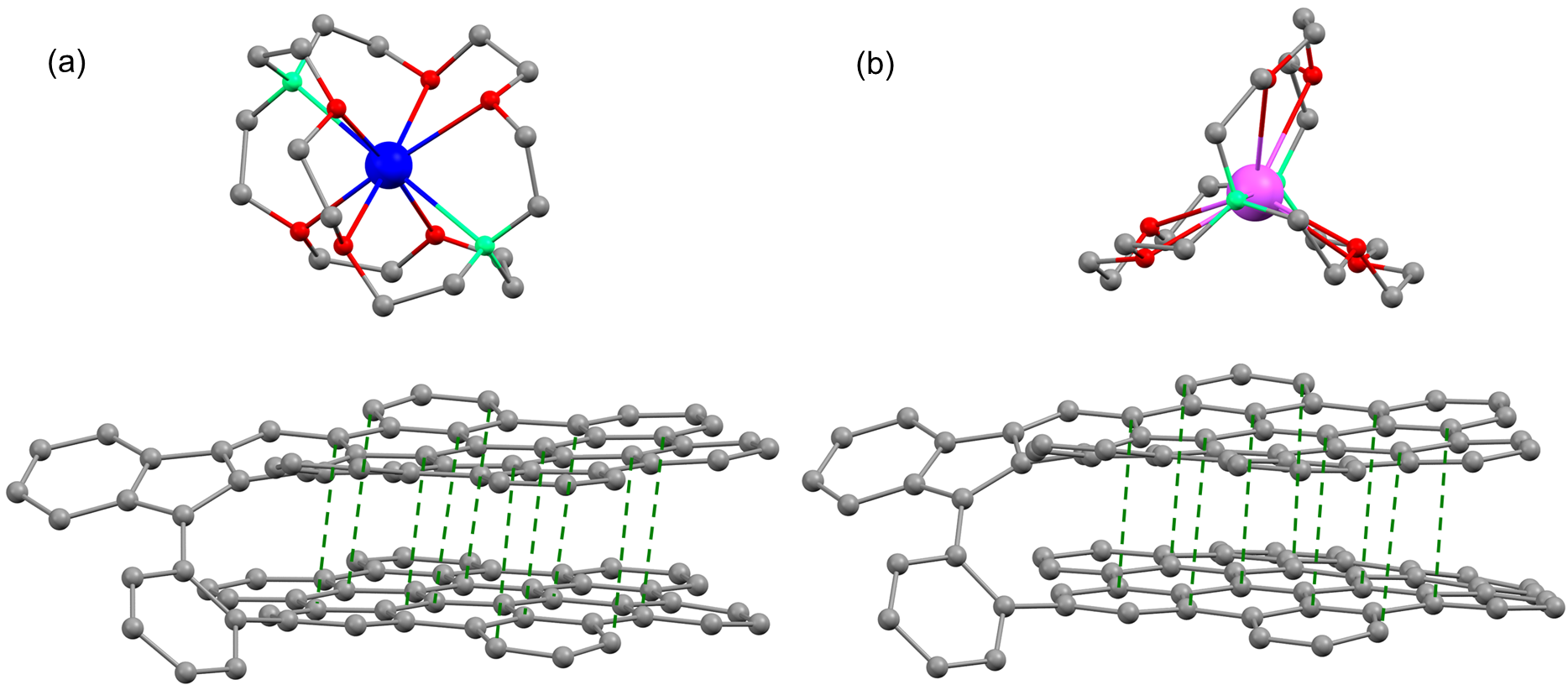


**Figure S10.** π–π interactions between the two HBC units (3.34(2)–3.66(2) Å) in **1** (a) and (3.43(2)–3.64(2) Å) in **2** (b) are shown in green, ball-and-stick models. H-atoms and tert-butyl groups are omitted for structure clarity. Color scheme used: C gray, O red, Na blue, K dark orchid, N spring green.


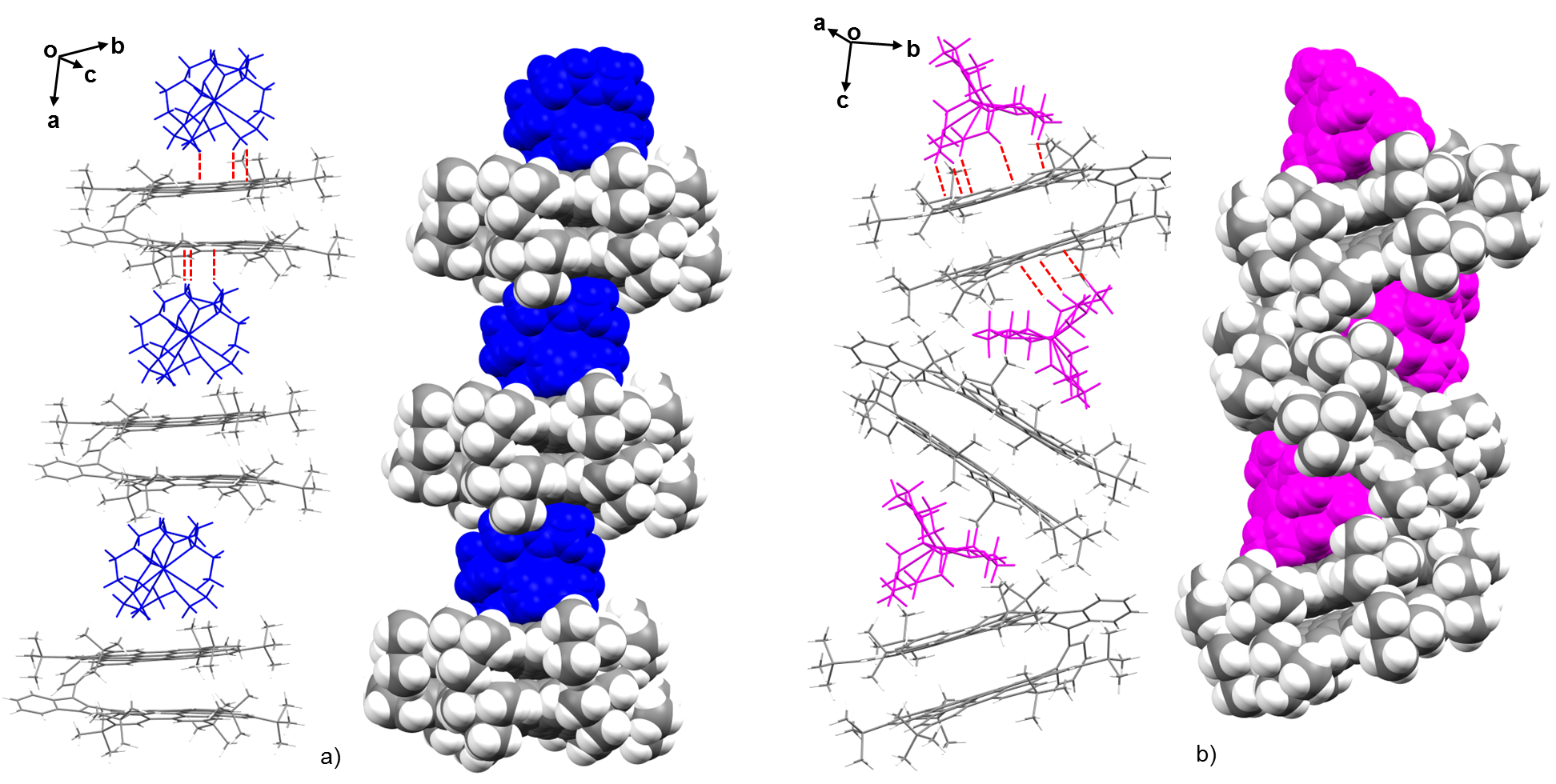


**Figure S11.** 1D column packing in (a) **1** and (b) **2**, capped-stick and space-filling models. The cationic moieties are highlighted in blue and pink for clarity. The C–H···π interactions (2.547(14)–2.800(14) Å) in **1** and (2.418(13)–2.879(13) Å) in **2** between the cationic moieties and the anionic cores are shown in red.

**Table S2.** Selected C–C bond length distances (Å) in **1** and **2**, along with a labeling scheme, *t*-butyl groups and hydrogen atoms are omitted for clarity.


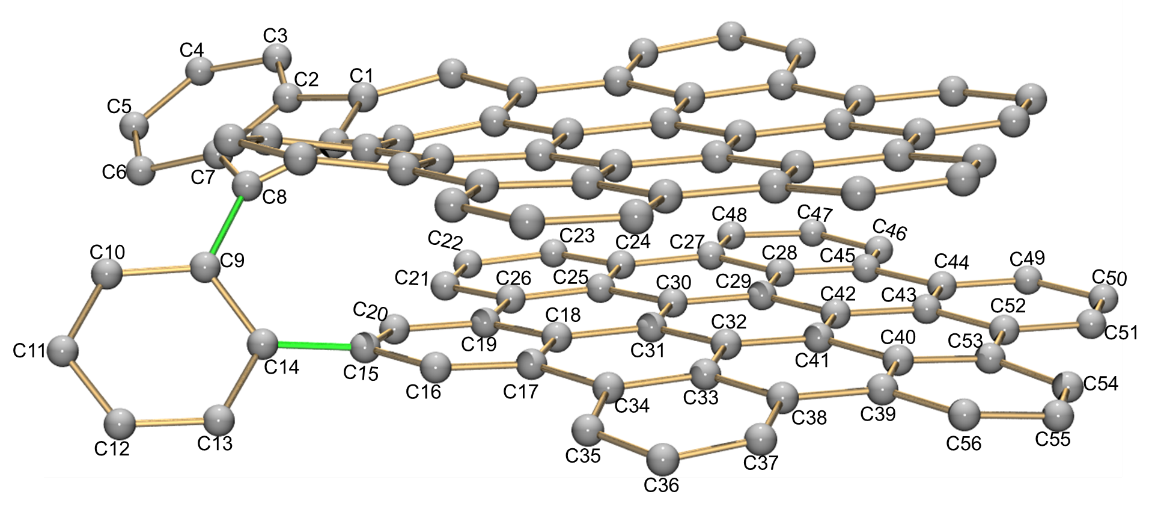


|  | **1** | **2** |  | **1** | **2** |
| --- | --- | --- | --- | --- | --- |
| C1–C2 | 1.445(4) | 1.442(3) | C19–C20 | 1.372(5) | 1.395(3) |
| C2–C3 | 1.386(5) | 1.398(3) | C21–C22 | 1.394(5) | 1.398(3) |
| C3–C4 | 1.386(5) | 1.386(3) | C22–C23 | 1.379(5) | 1.388(3) |
| C4–C5 | 1.386(6) | 1.387(3) | C23–C24 | 1.400(5) | 1.386(3) |
| C5–C6 | 1.397(5) | 1.388(3) | C24–C25 | 1.417(5) | 1.425(3) |
| C6–C7 | 1.413(5) | 1.406(3) | C25–C26 | 1.403(5) | 1.410(3) |
| C7–C8 | 1.446(4) | 1.442(3) | C21–C26 | 1.411(4) | 1.400(3) |
| C8–C9 | 1.471(4) | 1.470(3) | C27–C28 | 1.419(5) | 1.418(3) |
| C9–C10 | 1.392(5) | 1.406(3) | C28–C29 | 1.453(5) | 1.446(3) |
| C10–C11 | 1.397(5) | 1.394(3) | C29–C30 | 1.401(5) | 1.419(3) |
| C11–C12 | 1.383(5) | 1.371(4) | C25–C30 | 1.457(4) | 1.441(3) |
| C12–C13 | 1.375(6) | 1.379(3) | C30–C31 | 1.419(4) | 1.418(3) |
| C13–C14 | 1.402(5) | 1.385(3) | C31–C32 | 1.427(4) | 1.411(3) |
| C9–C14 | 1.429(4) | 1.410(3) | C32–C33 | 1.441(4) | 1.453(2) |
| C14–C15 | 1.480(5) | 1.494(3) | C33–C34 | 1.417(4) | 1.419(3) |
| C15–C16 | 1.400(4) | 1.387(3) | C17–C34 | 1.421(4) | 1.463(3) |
| C16–C17 | 1.384(5) | 1.399(3) | C34–C35 | 1.389(4) | 1.396(3) |
| C17–C18 | 1.421(4) | 1.426(3) | C35–C36 | 1.396(4) | 1.398(3) |
| C18–C19 | 1.430(4) | 1.416(3) | C36–C37 | 1.389(5) | 1.382(3) |
| C37–C38 | 1.399(4) | 1.399(2) | C47–C48 | 1.377(6) | 1.389(4) |
| C33–C38 | 1.425(4) | 1.409(3) | C49–C50 | 1.398(5) | 1.386(3) |
| C38–C39 | 1.458(5) | 1.464(3) | C50–C51 | 1.383(5) | 1.398(3) |
| C39–C40 | 1.424(4) | 1.423(2) | C51–C52 | 1.393(5) | 1.391(3) |
| C40–C41 | 1.454(4) | 1.439(3) | C43–C52 | 1.431(5) | 1.414(3) |
| C32–C41 | 1.404(5) | 1.413(3) | C52–C53 | 1.463(5) | 1.472(3) |
| C41–C42 | 1.415(5) | 1.421(2) | C40–C53 | 1.404(5) | 1.415(3) |
| C29–C42 | 1.424(4) | 1.407(3) | C53–C54 | 1.405(4) | 1.386(3) |
| C42–C43 | 1.438(5) | 1.449(3) | C54–C55 | 1.383(5) | 1.392(3) |
| C43–C44 | 1.418(5) | 1.421(3) | C55–C56 | 1.384(5) | 1.386(3) |
| C44–C45 | 1.475(5) | 1.461(3) | C39–C56 | 1.406(4) | 1.401(3) |
| C28–C45 | 1.403(5) | 1.417(3) | C44–C49 | 1.384(5) | 1.395(3) |
| C45–C46 | 1.400(5) | 1.397(3) | C18–C31 | 1.433(5) | 1.443(3) |
| C46–C47 | 1.385(5) | 1.380(3) | C27–C48 | 1.399(5) | 1.401(3) |

*C–C bond distances are averaged due to symmetry.

**Table S3.** Selected dihedral angles (°) in **1** and **2**, along with a labeling scheme, *t*-butyl groups and hydrogen atoms are omitted for clarity.


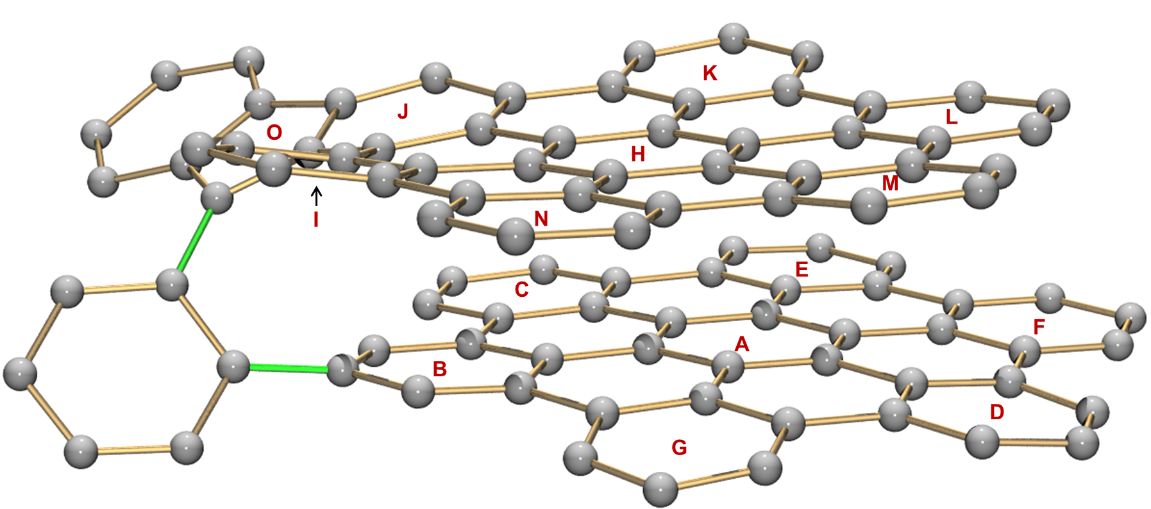


| **Angle** | **1** | **2** | **Angle** | **1** | **2** | **Angle** | **1** | **2** |
| --- | --- | --- | --- | --- | --- | --- | --- | --- |
| A/B | 1.63 | 0.89 | D/F | 3.94 | 2.90 | I/N | 8.33 | 6.09 |
| A/C | 3.24 | 2.50 | D/G | 9.70 | 14.05 | I/O | 33.44 | 39.20 |
| A/D | 1.93 | 6.68 | E/F | 3.67 | 6.03 | J/K | 5.88 | 9.50 |
| A/E | 1.66 | 8.78 | E/G | 7.65 | 15.94 | J/L | 9.13 | 8.96 |
| A/F | 5.10 | 3.83 | F/G | 9.46 | 11.31 | J/M | 11.72 | 11.72 |
| A/G | 8.34 | 7.52 | H/I | 11.71 | 13.48 | J/N | 14.13 | 19.63 |
| B/C | 4.08 | 2.45 | H/J | 10.81 | 12.08 | J/O | 15.16 | 14.52 |
| B/D | 1.39 | 7.57 | H/K | 7.62 | 6.06 | K/L | 6.46 | 5.25 |
| B/E | 2.77 | 9.55 | H/L | 1.72 | 3.18 | K/M | 10.14 | 9.60 |
| B/F | 5.32 | 4.73 | H/M | 3.47 | 4.72 | K/N | 10.20 | 12.90 |
| B/G | 9.96 | 6.64 | H/N | 3.44 | 7.64 | K/O | 20.57 | 23.52 |
| C/D | 5.05 | 7.32 | H/O | 25.73 | 26.57 | L/M | 3.75 | 4.46 |
| C/E | 4.07 | 10.70 | I/J | 22.46 | 25.12 | L/N | 5.16 | 10.68 |
| C/F | 8.23 | 4.95 | I/K | 18.20 | 18.97 | L/O | 24.01 | 23.41 |
| C/G | 8.15 | 7.00 | I/L | 13.43 | 16.20 | M/N | 5.34 | 9.27 |
| D/E | 2.07 | 5.75 | I/M | 12.32 | 13.81 | M/N | 25.77 | 25.41 |

**Table S4.** Selected torsion angles (°) in **1** and **2**, along with a labeling scheme, *t*-butyl groups and hydrogen atoms are omitted for clarity.


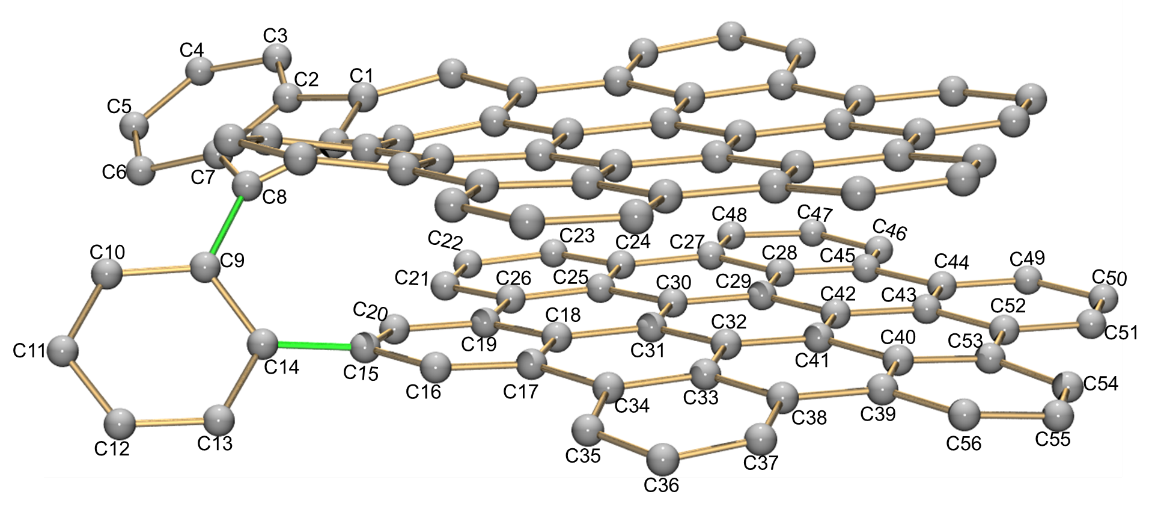


| **Angle** | **1** | **2** | **Angle** | **1** | **2** |
| --- | --- | --- | --- | --- | --- |
| C16–C17–C34–C35 | 9.89 | 3.35 | C37–C38–C39–56 | 4.74 | 2.74 |
| C20–C19–C26–C21 | 3.08 | 7.73 | C13–C14–C15–16 | 50.8 | 50.2 |
| C23–C24–C27–C48 | 0.19 | 5.18 | C8–C9–C14–C15 | 6.70 | 0.99 |
| C46–C44–C49–C50 | 3.03 | 5.06 | C10–C9–C8–C7 | 53.1 | 55.2 |
| C51–C52–C43–C54 | 4.00 | 1.85 | C9–C8–C7–C6 | 15.7 | 18.0 |

# **VI. Computational Details**

All the calculations reported in this paper were obtained with the GAUSSIAN 09 suite of programs.^[8]^ Electron correlation was partially taken into account using the (u)B3LYP^[9]^ functional in conjuction with the D3 dispersion correction suggested by Grimme *et al.*^[10]^ and the double-ζ quality plus polarization functions def2-SVP^[11]^ basis set for all atoms. All species were characterized by frequency calculations,^[12]^ and have positive definite Hessian matrices. This level is denoted B3LYP-D3/def2-SVP, which was proven to provide good results for strongly related systems.^[13]^ Nuclear Independent Chemical Shift (NICS)^[14]^ values were computed using the gauge-independent atomic orbital (GIAO)^[15]^ method at the same DFT level.

Cartesian coordinates (in Å) and total energies (in a.u., ZPVE included) of all the stationary points discussed in the text. All calculations have been performed at the B3LYP-D3/def2-SVP level.

**1M:** E= -4110.031213

C 0.617685000 2.288844000 1.231151000

C 0.047080000 1.016895000 1.513363000

C -1.364318000 0.857105000 1.576720000

C -2.213433000 1.979605000 1.379466000

C -1.647727000 3.259706000 1.121530000

C -0.235255000 3.411572000 1.032665000

C 0.908048000 -0.104810000 1.789522000

C 2.319971000 0.055755000 1.758439000

C 3.130383000 -0.957437000 2.288192000

H 4.197269000 -0.779639000 2.394215000

C 2.605308000 -2.186056000 2.691419000

C 1.229366000 -2.390795000 2.561792000

H 0.827156000 -3.364541000 2.842214000

C 0.363384000 -1.373355000 2.136507000

C -1.930988000 -0.449957000 1.820375000

C -1.083580000 -1.573108000 2.052776000

C -1.655557000 -2.845133000 2.187179000

H -1.013167000 -3.718793000 2.298330000

C -3.037247000 -3.046275000 2.145394000

C -3.859307000 -1.933033000 1.971404000

H -4.934451000 -2.099095000 1.926629000

C -3.342294000 -0.636941000 1.811236000

C -3.647859000 1.812087000 1.418346000

C -4.218122000 0.521059000 1.620207000

C -5.614810000 0.385866000 1.617544000

H -6.063867000 -0.594714000 1.768007000

C -6.466685000 1.469439000 1.405231000

C -5.897462000 2.729499000 1.212963000

H -6.570048000 3.570522000 1.046271000

C -4.508584000 2.931037000 1.223576000

C -2.510035000 4.405094000 0.939045000

C -3.928071000 4.259733000 1.013036000

C -4.737355000 5.393821000 0.857516000

H -5.821243000 5.303211000 0.926668000

C -4.207301000 6.660837000 0.604703000

C -2.822318000 6.788461000 0.513744000

H -2.415066000 7.778037000 0.309971000

C -1.956863000 5.693162000 0.680822000

C 0.333664000 4.705247000 0.734966000

C -0.503352000 5.848577000 0.581203000

C 0.088932000 7.098085000 0.331018000

H -0.535725000 7.983805000 0.218787000

C 1.471002000 7.260376000 0.227471000

C 2.279045000 6.127569000 0.347459000

H 3.359217000 6.255876000 0.269907000

C 1.743531000 4.852092000 0.580902000

C 2.049420000 2.418236000 1.051994000

C 2.601204000 3.667745000 0.640346000

C 3.933050000 3.715392000 0.204546000

H 4.353901000 4.638157000 -0.191891000

C 4.663377000 2.539885000 0.083103000

C 4.171399000 1.312279000 0.571663000

C 2.891095000 1.263131000 1.168586000

C 5.842799000 2.308965000 -0.756215000

C 6.664889000 3.198893000 -1.455636000

H 6.541272000 4.278739000 -1.341868000

C 7.638140000 2.682447000 -2.317842000

H 8.286635000 3.364370000 -2.873617000

C 7.783400000 1.296836000 -2.483630000

H 8.540960000 0.909877000 -3.169613000

C 6.968473000 0.404981000 -1.774102000

H 7.083935000 -0.675609000 -1.887434000

C 6.002960000 0.922271000 -0.916632000

C 5.057398000 0.166853000 0.029940000

C 6.034132000 -0.523739000 0.994010000

C 6.943965000 0.057029000 1.871382000

H 6.981611000 1.142674000 1.989098000

C 7.805202000 -0.777730000 2.595584000

H 8.519756000 -0.340696000 3.297451000

C 7.760719000 -2.169580000 2.424456000

H 8.444243000 -2.806213000 2.991853000

C 6.843107000 -2.750160000 1.542202000

H 6.797641000 -3.835678000 1.424434000

C 5.974467000 -1.918006000 0.827967000

C 4.830563000 -2.228365000 -0.034891000

C 4.184762000 -3.450846000 -0.173612000

H 4.659626000 -4.344807000 0.228002000

C 2.861079000 -3.492247000 -0.635005000

C 2.232778000 -2.280518000 -1.049666000

C 2.995407000 -1.071187000 -1.149951000

C 4.264502000 -1.035666000 -0.530079000

C 0.798361000 -2.245275000 -1.245091000

C 0.147641000 -1.010843000 -1.521582000

C -1.270317000 -0.940276000 -1.580244000

C -2.045236000 -2.116565000 -1.390761000

C -1.398134000 -3.361849000 -1.156298000

C 0.020523000 -3.423442000 -1.065470000

C 0.935285000 0.164559000 -1.792672000

C 2.353984000 0.096367000 -1.747942000

C 3.102195000 1.159369000 -2.270880000

H 4.179869000 1.052415000 -2.362050000

C 2.501421000 2.349160000 -2.685403000

C 1.113400000 2.463328000 -2.572007000

H 0.651254000 3.408026000 -2.859683000

C 0.311670000 1.393351000 -2.149232000

C -1.919514000 0.328852000 -1.816624000

C -1.145693000 1.499965000 -2.070112000

C -1.798773000 2.730072000 -2.224162000

H -1.215601000 3.641352000 -2.357676000

C -3.190468000 2.842649000 -2.179033000

C -3.938762000 1.684748000 -1.968476000

H -5.022605000 1.782600000 -1.927650000

C -3.339296000 0.427980000 -1.783695000

C -3.486662000 -2.036672000 -1.398261000

C -4.138102000 -0.777478000 -1.551448000

C -5.536383000 -0.720938000 -1.457784000

H -6.046481000 0.237455000 -1.540549000

C -6.313227000 -1.855754000 -1.222517000

C -5.667544000 -3.088154000 -1.110176000

H -6.280181000 -3.970532000 -0.925885000

C -4.271992000 -3.209368000 -1.203414000

C -2.184501000 -4.564074000 -1.002942000

C -3.607199000 -4.506732000 -1.068802000

C -4.343305000 -5.698882000 -0.974888000

H -5.429779000 -5.673364000 -1.054063000

C -3.732162000 -6.937265000 -0.781642000

C -2.340070000 -6.976365000 -0.674178000

H -1.870715000 -7.945921000 -0.508132000

C -1.548300000 -5.822658000 -0.783755000

C 0.671726000 -4.680892000 -0.780662000

C -0.088077000 -5.880850000 -0.661002000

C 0.583809000 -7.090060000 -0.416507000

H 0.019089000 -8.017506000 -0.325288000

C 1.971986000 -7.159051000 -0.287533000

C 2.703017000 -5.972522000 -0.377042000

H 3.787919000 -6.027662000 -0.280473000

C 2.085536000 -4.733174000 -0.603748000

C 2.670298000 -8.481896000 -0.086855000

H 3.566012000 -8.374885000 0.544735000

H 2.007666000 -9.224578000 0.383325000

H 3.001615000 -8.902362000 -1.052615000

C -4.542231000 -8.208648000 -0.711551000

H -4.491288000 -8.761607000 -1.665674000

H -4.164973000 -8.883556000 0.073259000

H -5.603644000 -8.005168000 -0.504352000

C -7.807043000 -1.742669000 -1.043320000

H -8.227098000 -0.925065000 -1.649519000

H -8.323977000 -2.674359000 -1.319583000

H -8.059372000 -1.525806000 0.009400000

C -7.961812000 1.276366000 1.337662000

H -8.283557000 1.058625000 0.304139000

H -8.290009000 0.431332000 1.962404000

H -8.504797000 2.175783000 1.666563000

C -5.123308000 7.845968000 0.420872000

H -5.755406000 7.722694000 -0.474957000

H -5.803642000 7.960941000 1.280783000

H -4.560771000 8.784517000 0.307122000

C -3.855253000 4.182033000 -2.374724000

H -3.870789000 4.460459000 -3.442988000

H -4.893428000 4.174652000 -2.012867000

H -3.318971000 4.975839000 -1.833208000

C 2.082107000 8.624900000 0.021911000

H 2.381078000 9.071459000 0.986497000

H 2.985888000 8.573156000 -0.605241000

H 1.374591000 9.319955000 -0.455496000

C 3.317164000 3.499904000 -3.217708000

H 4.392357000 3.272035000 -3.216744000

H 3.019891000 3.757288000 -4.248098000

H 3.160858000 4.400330000 -2.600407000

C -3.613322000 -4.430986000 2.304092000

H -4.643842000 -4.485932000 1.924700000

H -3.016805000 -5.176055000 1.756318000

H -3.627693000 -4.731688000 3.366373000

C 3.488643000 -3.282120000 3.231244000

H 3.199364000 -3.558076000 4.259123000

H 3.396044000 -4.190658000 2.613027000

H 4.546815000 -2.985396000 3.239800000

**1M·^-^:** E= -4110.077945

C -0.714750000 2.328939000 -1.202299000

C -0.079163000 1.086532000 -1.478762000

C 1.343140000 0.997322000 -1.526733000

C 2.135577000 2.165702000 -1.315793000

C 1.504874000 3.415995000 -1.075384000

C 0.079743000 3.498989000 -1.008000000

C -0.880700000 -0.075867000 -1.757966000

C -2.303060000 0.009315000 -1.718149000

C -3.061727000 -1.058547000 -2.230096000

H -4.136803000 -0.938777000 -2.334791000

C -2.471388000 -2.256811000 -2.624545000

C -1.081555000 -2.382616000 -2.516481000

H -0.629679000 -3.334980000 -2.794893000

C -0.268746000 -1.315838000 -2.106413000

C 1.975282000 -0.269504000 -1.776195000

C 1.189052000 -1.434894000 -2.033728000

C 1.831908000 -2.668523000 -2.200326000

H 1.239278000 -3.573998000 -2.331531000

C 3.224159000 -2.794072000 -2.174255000

C 3.983514000 -1.644497000 -1.961295000

H 5.066589000 -1.752991000 -1.921026000

C 3.398629000 -0.384631000 -1.754227000

C 3.575730000 2.068573000 -1.316337000

C 4.213135000 0.803786000 -1.501392000

C 5.611917000 0.727775000 -1.411921000

H 6.111726000 -0.232200000 -1.528220000

C 6.402780000 1.852298000 -1.149704000

C 5.777835000 3.085335000 -1.000614000

H 6.401637000 3.954239000 -0.791612000

C 4.375699000 3.227632000 -1.087260000

C 2.305518000 4.601491000 -0.884913000

C 3.731249000 4.520735000 -0.914815000

C 4.485036000 5.701741000 -0.747756000

H 5.573048000 5.659766000 -0.790435000

C 3.888397000 6.936451000 -0.520888000

C 2.490807000 7.001775000 -0.468656000

H 2.032924000 7.972729000 -0.277531000

C 1.684202000 5.869064000 -0.655130000

C -0.554144000 4.760436000 -0.747324000

C 0.220754000 5.953557000 -0.604820000

C -0.440703000 7.175841000 -0.412617000

H 0.136466000 8.096299000 -0.318845000

C -1.833848000 7.271060000 -0.346643000

C -2.579632000 6.095516000 -0.442766000

H -3.667202000 6.167003000 -0.393180000

C -1.975548000 4.840542000 -0.621486000

C -2.149141000 2.386224000 -1.032025000

C -2.768993000 3.614288000 -0.649453000

C -4.104573000 3.602884000 -0.214785000

H -4.568842000 4.507873000 0.174572000

C -4.766330000 2.387453000 -0.066596000

C -4.220052000 1.182087000 -0.548338000

C -2.932647000 1.185437000 -1.144212000

C -5.915572000 2.104243000 0.796393000

C -6.762587000 2.956402000 1.514698000

H -6.682453000 4.040907000 1.404773000

C -7.698652000 2.397426000 2.392865000

H -8.363627000 3.051483000 2.963724000

C -7.783514000 1.007139000 2.557635000

H -8.511317000 0.586404000 3.256548000

C -6.940885000 0.152662000 1.831953000

H -7.002930000 -0.932508000 1.945124000

C -6.013082000 0.711451000 0.960226000

C -5.047462000 -0.000312000 0.000036000

C -6.013011000 -0.712185000 -0.960144000

C -6.940893000 -0.153501000 -1.831856000

H -7.003065000 0.931661000 -1.945022000

C -7.783432000 -1.008074000 -2.557527000

H -8.511296000 -0.587422000 -3.256428000

C -7.698407000 -2.398352000 -2.392763000

H -8.363316000 -3.052484000 -2.963614000

C -6.762262000 -2.957222000 -1.514613000

H -6.682001000 -4.041717000 -1.404690000

C -5.915337000 -2.104966000 -0.796319000

C -4.766046000 -2.388046000 0.066648000

C -4.104131000 -3.603392000 0.214801000

H -4.568298000 -4.508428000 -0.174566000

C -2.768536000 -3.614636000 0.649431000

C -2.148840000 -2.386505000 1.032039000

C -2.932499000 -1.185821000 1.144253000

C -4.219907000 -1.182618000 0.548395000

C -0.714454000 -2.329037000 1.202307000

C -0.079025000 -1.086548000 1.478765000

C 1.343266000 -0.997152000 1.526714000

C 2.135850000 -2.165430000 1.315777000

C 1.505306000 -3.415807000 1.075388000

C 0.080186000 -3.498985000 1.008002000

C -0.880705000 0.075748000 1.757984000

C -2.303056000 -0.009619000 1.718192000

C -3.061853000 1.058138000 2.230149000

H -4.136913000 0.938238000 2.334858000

C -2.471663000 2.256486000 2.624585000

C -1.081854000 2.382471000 2.516498000

H -0.630092000 3.334893000 2.794895000

C -0.268909000 1.315795000 2.106421000

C 1.975250000 0.269757000 1.776167000

C 1.188873000 1.435041000 2.033725000

C 1.831574000 2.668748000 2.200349000

H 1.238830000 3.574144000 2.331589000

C 3.223809000 2.794473000 2.174276000

C 3.983308000 1.645004000 1.961266000

H 5.066371000 1.753637000 1.920996000

C 3.398582000 0.385068000 1.754168000

C 3.575990000 -2.068109000 1.316289000

C 4.213233000 -0.803237000 1.501290000

C 5.612006000 -0.727039000 1.411725000

H 6.111687000 0.233008000 1.527971000

C 6.402999000 -1.851455000 1.149468000

C 5.778211000 -3.084591000 1.000482000

H 6.402109000 -3.953420000 0.791450000

C 4.376106000 -3.227072000 1.087238000

C 2.306102000 -4.601211000 0.884982000

C 3.731822000 -4.520275000 0.914928000

C 4.485763000 -5.701204000 0.748066000

H 5.573768000 -5.659090000 0.790813000

C 3.889284000 -6.936027000 0.521378000

C 2.491710000 -7.001523000 0.469055000

H 2.033962000 -7.972576000 0.278114000

C 1.684952000 -5.868875000 0.655260000

C -0.553535000 -4.760507000 0.747275000

C 0.221517000 -5.953525000 0.604747000

C -0.439775000 -7.175860000 0.412302000

H 0.137518000 -8.096225000 0.318379000

C -1.832902000 -7.271246000 0.346217000

C -2.578840000 -6.095812000 0.442472000

H -3.666396000 -6.167444000 0.392820000

C -1.974926000 -4.840781000 0.621369000

C -2.508010000 -8.614888000 0.207675000

H -3.513171000 -8.521293000 -0.232753000

H -1.921962000 -9.299192000 -0.427131000

H -2.626135000 -9.107079000 1.190026000

C 4.712650000 -8.187528000 0.331359000

H 4.482416000 -8.940146000 1.105269000

H 4.503372000 -8.659235000 -0.644208000

H 5.792025000 -7.976979000 0.377910000

C 7.896234000 -1.703963000 0.984740000

H 8.322891000 -1.035152000 1.750422000

H 8.414297000 -2.673255000 1.051453000

H 8.143389000 -1.261127000 0.003567000

C 7.896062000 1.704967000 -0.985265000

H 8.143381000 1.260649000 -0.004809000

H 8.322860000 1.037453000 -1.752009000

H 8.413861000 2.674496000 -1.050523000

C 4.711593000 8.188014000 -0.330549000

H 4.502286000 8.659406000 0.645165000

H 5.790997000 7.977633000 -0.377203000

H 4.481216000 8.940821000 -1.104232000

C 3.881258000 4.135402000 2.388893000

H 4.028687000 4.339402000 3.465145000

H 4.865782000 4.179530000 1.900101000

H 3.271416000 4.952035000 1.973960000

C -2.509178000 8.614614000 -0.208318000

H -2.628203000 9.106233000 -1.190846000

H -3.513984000 8.521001000 0.232926000

H -1.922821000 9.299400000 0.425673000

C -3.297885000 3.409685000 3.137067000

H -4.368111000 3.159983000 3.171399000

H -2.979995000 3.712950000 4.149306000

H -3.178883000 4.288548000 2.481281000

C 3.881783000 -4.134925000 -2.388810000

H 4.866303000 -4.178903000 -1.899996000

H 3.272038000 -4.951620000 -1.973856000

H 4.029263000 -4.338951000 -3.465050000

C -3.297448000 -3.410130000 -3.137017000

H -2.979531000 -3.713339000 -4.149264000

H -3.178294000 -4.288977000 -2.481239000

H -4.367714000 -3.160592000 -3.171326000

**TS:** E= -4110.036514 (i = -135 cm-1)

C 0.235909000 -1.044663000 -1.632484000

C -0.062493000 0.344100000 -1.734482000

C -1.414711000 0.785162000 -1.725279000

C -2.473095000 -0.162911000 -1.664360000

C -2.178636000 -1.553307000 -1.603775000

C -0.825051000 -1.992537000 -1.563163000

C 1.009366000 1.299959000 -1.878688000

C 2.362199000 0.859568000 -1.927504000

C 3.378425000 1.780239000 -2.219642000

H 4.402802000 1.414094000 -2.288079000

C 3.102062000 3.134459000 -2.411635000

C 1.782300000 3.574027000 -2.272550000

H 1.578229000 4.637839000 -2.400511000

C 0.728286000 2.689831000 -1.997949000

C -1.708666000 2.199818000 -1.754491000

C -0.650259000 3.153364000 -1.836146000

C -0.956069000 4.517677000 -1.738241000

H -0.148870000 5.249880000 -1.714922000

C -2.268313000 4.975950000 -1.613456000

C -3.300569000 4.039635000 -1.603433000

H -4.320456000 4.406676000 -1.498639000

C -3.054209000 2.658813000 -1.669046000

C -3.845239000 0.287714000 -1.650857000

C -4.146011000 1.682692000 -1.639238000

C -5.487659000 2.088657000 -1.585961000

H -5.731036000 3.150225000 -1.554537000

C -6.540544000 1.173538000 -1.560581000

C -6.236939000 -0.186238000 -1.599277000

H -7.065801000 -0.892510000 -1.572765000

C -4.914315000 -0.655508000 -1.629370000

C -3.252755000 -2.518236000 -1.569919000

C -4.611603000 -2.087715000 -1.601412000

C -5.634345000 -3.050652000 -1.566868000

H -6.677527000 -2.737072000 -1.594130000

C -5.366309000 -4.416482000 -1.486690000

C -4.032410000 -4.826870000 -1.447477000

H -3.829995000 -5.895379000 -1.373990000

C -2.966644000 -3.914951000 -1.491877000

C -0.526038000 -3.400631000 -1.446225000

C -1.574444000 -4.364489000 -1.424806000

C -1.249918000 -5.727103000 -1.319123000

H -2.039414000 -6.477409000 -1.303866000

C 0.068711000 -6.165721000 -1.209012000

C 1.088829000 -5.213862000 -1.221787000

H 2.119024000 -5.562974000 -1.149831000

C 0.828997000 -3.838771000 -1.337530000

C 1.611947000 -1.481228000 -1.557719000

C 1.909732000 -2.857831000 -1.345389000

C 3.239386000 -3.224601000 -1.083670000

H 3.472465000 -4.250151000 -0.799387000

C 4.258746000 -2.269995000 -1.041770000

C 4.003605000 -0.945198000 -1.399664000

C 2.682838000 -0.534022000 -1.647767000

C 5.571324000 -2.532835000 -0.433254000

C 6.179801000 -3.790166000 -0.306104000

H 5.701597000 -4.668593000 -0.746976000

C 7.393644000 -3.925568000 0.369820000

H 7.861998000 -4.909737000 0.460989000

C 8.006046000 -2.798755000 0.930565000

H 8.954170000 -2.897040000 1.466810000

C 7.406126000 -1.542311000 0.799081000

H 7.884545000 -0.658324000 1.227662000

C 6.184132000 -1.391525000 0.125638000

C 5.572047000 -0.035443000 -0.061521000

C 6.484205000 0.954241000 -0.648529000

C 7.578272000 0.773376000 -1.512768000

H 7.854413000 -0.231080000 -1.842354000

C 8.300607000 1.887911000 -1.942165000

H 9.152249000 1.753088000 -2.615769000

C 7.949790000 3.186186000 -1.519117000

H 8.533527000 4.045462000 -1.861275000

C 6.857232000 3.377931000 -0.668616000

H 6.576651000 4.384832000 -0.346660000

C 6.123274000 2.267232000 -0.239986000

C 4.916912000 2.141580000 0.555515000

C 4.018077000 3.135999000 0.893184000

H 4.293214000 4.171875000 0.694933000

C 2.713793000 2.811084000 1.304452000

C 2.381265000 1.429907000 1.483621000

C 3.381923000 0.416791000 1.378125000

C 4.627696000 0.745893000 0.753165000

C 0.995756000 1.052970000 1.669154000

C 0.637100000 -0.321209000 1.768351000

C -0.730115000 -0.708799000 1.813407000

C -1.748939000 0.279623000 1.770376000

C -1.397357000 1.658533000 1.723326000

C -0.028392000 2.045913000 1.669843000

C 1.670476000 -1.315349000 1.881049000

C 3.038309000 -0.923882000 1.811839000

C 4.023045000 -1.846142000 2.206893000

H 5.053742000 -1.509443000 2.284866000

C 3.711213000 -3.168943000 2.515390000

C 2.378525000 -3.583811000 2.408632000

H 2.144575000 -4.629314000 2.614888000

C 1.346716000 -2.682854000 2.103498000

C -1.074107000 -2.109249000 1.891546000

C -0.053083000 -3.098911000 2.008025000

C -0.414898000 -4.454736000 2.005355000

H 0.361865000 -5.218898000 2.026850000

C -1.745210000 -4.864549000 1.923428000

C -2.739228000 -3.887201000 1.858846000

H -3.774582000 -4.219057000 1.787540000

C -2.438524000 -2.517095000 1.843589000

C -3.137237000 -0.117054000 1.767370000

C -3.491004000 -1.499650000 1.777494000

C -4.846555000 -1.853208000 1.720726000

H -5.133215000 -2.903745000 1.688083000

C -5.862086000 -0.898560000 1.669287000

C -5.506940000 0.448821000 1.681594000

H -6.306338000 1.185895000 1.623181000

C -4.167626000 0.866817000 1.725400000

C -2.432832000 2.665516000 1.714301000

C -3.808691000 2.287242000 1.709947000

C -4.791339000 3.287909000 1.669950000

H -5.845507000 3.013388000 1.642095000

C -4.468523000 4.645267000 1.652460000

C -3.122602000 5.005108000 1.686554000

H -2.878576000 6.066524000 1.666764000

C -2.092505000 4.051163000 1.702203000

C 0.322827000 3.445519000 1.605848000

C -0.685082000 4.450184000 1.661545000

C -0.306597000 5.804419000 1.635315000

H -1.063377000 6.585905000 1.698537000

C 1.030396000 6.193458000 1.533013000

C 2.007839000 5.203172000 1.438997000

H 3.049805000 5.513546000 1.355282000

C 1.691394000 3.831528000 1.462364000

C 1.413733000 7.654404000 1.552618000

H 2.302768000 7.847447000 0.931276000

H 0.595724000 8.293956000 1.184435000

H 1.655065000 7.989415000 2.577375000

C -5.546779000 5.694500000 1.531451000

H -5.776689000 5.900294000 0.469840000

H -6.485687000 5.373486000 2.009904000

H -5.239914000 6.649310000 1.986656000

C -7.303538000 -1.331941000 1.568637000

H -7.435802000 -2.054221000 0.746097000

H -7.642228000 -1.830784000 2.493299000

H -7.973246000 -0.479038000 1.380589000

C -7.967875000 1.644895000 -1.425574000

H -8.680869000 0.912074000 -1.834795000

H -8.230683000 1.796531000 -0.362622000

H -8.131519000 2.605641000 -1.939123000

C -6.482783000 -5.433153000 -1.463718000

H -6.599493000 -5.920775000 -2.447824000

H -6.284917000 -6.232557000 -0.730933000

H -7.448980000 -4.970970000 -1.208260000

C -2.112592000 -6.327879000 1.905829000

H -1.268518000 -6.950272000 1.573496000

H -2.415465000 -6.678842000 2.908664000

H -2.957783000 -6.515200000 1.224508000

C 0.391522000 -7.630160000 -1.034217000

H 1.272140000 -7.925129000 -1.627520000

H 0.624563000 -7.858940000 0.021299000

H -0.451508000 -8.273242000 -1.331027000

C 4.781427000 -4.147231000 2.933622000

H 5.782336000 -3.695515000 2.889615000

H 4.608649000 -4.517469000 3.959439000

H 4.791910000 -5.025884000 2.267225000

C -2.541633000 6.451083000 -1.458419000

H -3.605612000 6.650294000 -1.259783000

H -1.956234000 6.864108000 -0.620519000

H -2.252410000 7.013689000 -2.362802000

C 4.202263000 4.121696000 -2.707574000

H 3.909208000 4.825409000 -3.504392000

H 4.431883000 4.720572000 -1.809799000

H 5.134343000 3.619809000 -3.001269000

**INT:** E= -4110.036514

C 4.224415000 -4.532397000 -1.317093000

H 4.006628000 -5.595304000 -1.216484000

C 3.472483000 -2.222132000 -1.533905000

C 1.241461000 1.303802000 1.756821000

C -0.854755000 -0.612763000 1.716056000

C 3.171191000 -3.614165000 -1.461870000

C 2.310273000 2.277031000 1.770230000

C 5.559215000 -4.131722000 -1.280072000

C -0.622575000 -3.520542000 -1.530681000

C 1.053955000 -1.676718000 -1.617299000

C 2.409145000 -1.247259000 -1.606535000

C -1.919782000 -1.579669000 1.755406000

C -1.171717000 0.774837000 1.655733000

C 0.006172000 -0.717031000 -1.695124000

C 4.588286000 -2.311616000 1.839224000

H 4.843409000 -3.370089000 1.821599000

C 2.015737000 3.673347000 1.756729000

C 4.407858000 1.970810000 -1.587210000

C 5.151181000 -0.374198000 -1.554137000

C 3.673015000 1.857306000 1.789559000

C 5.844394000 -2.771149000 -1.400056000

H 6.890388000 -2.468288000 -1.371346000

C 0.500689000 -1.042903000 1.789364000

C 2.715781000 0.142995000 -1.639192000

C 1.550638000 -0.086010000 1.792054000

C 0.736442000 -3.086139000 -1.554022000

C 3.075342000 4.593087000 1.759913000

H 2.865016000 5.661630000 1.744323000

C 4.092642000 0.578962000 -1.600662000

C -0.114798000 1.735446000 1.698259000

C 3.240599000 -1.915834000 1.847394000

C 2.926442000 -0.525246000 1.824286000

C 1.666699000 1.102617000 -1.691223000

C -1.368909000 -1.151326000 -1.683668000

C 0.311353000 0.673309000 -1.735069000

C 5.630984000 -1.389239000 1.813657000

C -0.754584000 1.646778000 -1.809080000

C 4.835701000 -1.803279000 -1.510764000

C -2.546081000 1.201153000 1.479256000

C -8.284256000 3.426883000 -0.862824000

H -8.847419000 4.340980000 -1.071268000

C 5.752815000 2.362832000 -1.520090000

H 6.009340000 3.421577000 -1.489060000

C 0.623045000 4.118341000 1.710371000

C -2.424749000 -0.192808000 -1.749601000

C 0.803400000 -2.453273000 1.853557000

C 4.688242000 2.826268000 1.774305000

H 5.732909000 2.518104000 1.761827000

C -1.697571000 -2.529134000 -1.537967000

C -1.640882000 -2.963740000 1.926190000

C -0.468659000 3.041389000 -1.811956000

C -0.418359000 3.148705000 1.664988000

C -0.249224000 -3.415343000 1.903918000

C -2.837374000 2.602758000 1.413792000

C -5.098082000 2.066765000 0.725658000

C -6.285539000 -1.412003000 -0.251098000

C -3.577873000 0.228788000 1.299375000

C -0.897003000 -4.895473000 -1.500998000

H -1.931769000 -5.238164000 -1.498077000

C -6.378360000 2.305646000 0.108019000

C -1.027507000 5.920726000 1.605659000

C -1.776319000 3.583567000 1.564176000

C -8.075756000 0.999522000 -1.068206000

H -8.458932000 0.051974000 -1.452748000

C -3.035483000 -2.895151000 -1.338654000

H -3.271981000 -3.924797000 -1.075399000

C 3.325896000 2.957974000 -1.616752000

C 2.157588000 -2.900756000 1.868435000

C -1.535519000 3.949255000 -1.889880000

H -1.338695000 5.021106000 -1.869965000

C 3.989034000 0.427040000 1.812162000

C 5.313846000 -0.030202000 1.806860000

H 6.136391000 0.682849000 1.766196000

C 4.410384000 4.191815000 1.754693000

C -3.277606000 -1.146475000 1.650576000

C -3.730159000 -0.644539000 -1.616595000

C -2.110520000 1.223727000 -1.861605000

C -4.149558000 2.993753000 1.112429000

H -4.415957000 4.048568000 1.057831000

C 1.776832000 -4.057293000 -1.508025000

C 6.797308000 1.437857000 -1.480526000

C -7.082168000 3.488444000 -0.157835000

H -6.688685000 4.451285000 0.182115000

C 0.294983000 5.485642000 1.677872000

H 1.081085000 6.239363000 1.702945000

C -6.870403000 1.036375000 -0.329648000

C 0.115643000 -5.855412000 -1.482623000

C 1.439783000 -5.420761000 -1.494227000

H 2.225575000 -6.175698000 -1.488615000

C 1.972471000 2.516108000 -1.680992000

C 6.478970000 0.081170000 -1.509894000

H 7.299443000 -0.635039000 -1.483608000

C -5.931588000 -0.006752000 0.042775000

C 3.591771000 4.334564000 -1.563541000

H 4.619763000 4.689521000 -1.502205000

C -4.297855000 -2.067362000 1.936175000

H -5.325091000 -1.713515000 1.981603000

C -7.563891000 -1.867914000 0.149105000

H -8.198373000 -1.182405000 0.714457000

C 0.078162000 -4.780757000 1.917787000

H -0.717101000 -5.525832000 1.914505000

C -4.082503000 -1.954140000 -1.338025000

C -2.710288000 -3.857808000 2.114354000

H -2.513124000 -4.918773000 2.269813000

C 2.420579000 -4.278293000 1.899322000

H 3.449581000 -4.636476000 1.892800000

C 1.398573000 -5.227974000 1.912082000

C -8.768397000 2.181314000 -1.319568000

H -9.702086000 2.144693000 -1.889554000

C -2.041945000 4.964814000 1.555975000

H -3.072278000 5.316213000 1.496964000

C -4.833804000 0.642832000 0.728785000

C -4.034695000 -3.418029000 2.169657000

C -5.454871000 -2.336251000 -0.945316000

C 0.923800000 3.483805000 -1.713750000

C -3.141252000 2.168162000 -1.959588000

H -4.172906000 1.811899000 -1.983086000

C -8.025127000 -3.152530000 -0.130033000

H -9.019288000 -3.459637000 0.207348000

C 2.573058000 5.286435000 -1.552795000

C -2.868474000 3.535458000 -1.971134000

C 1.253172000 4.844812000 -1.635622000

H 0.460507000 5.591462000 -1.612072000

C -7.208457000 -4.043226000 -0.835502000

H -7.558074000 -5.050369000 -1.078381000

C -5.936581000 -3.628868000 -1.225637000

H -5.301602000 -4.316806000 -1.788700000

C 2.881756000 6.752780000 -1.382558000

H 2.778755000 7.047211000 -0.322767000

H 3.910834000 6.993773000 -1.691665000

H 2.190956000 7.385721000 -1.961803000

C -3.978239000 4.554919000 -2.027166000

H -4.967521000 4.082765000 -1.948909000

H -3.882679000 5.276573000 -1.198276000

H -3.943817000 5.134302000 -2.966292000

C 8.228130000 1.906303000 -1.367887000

H 8.439787000 2.289455000 -0.353815000

H 8.941341000 1.092201000 -1.568915000

H 8.441548000 2.727530000 -2.071621000

C 6.671775000 -5.128988000 -1.064042000

H 7.546344000 -4.903252000 -1.695524000

H 7.018993000 -5.109889000 -0.015291000

H 6.348276000 -6.157766000 -1.285039000

C 7.072456000 -1.830382000 1.760229000

H 7.579320000 -1.397792000 0.881540000

H 7.631187000 -1.496421000 2.651477000

H 7.161233000 -2.925220000 1.694308000

C 1.723684000 -6.700477000 1.872135000

H 1.960695000 -7.018665000 0.842108000

H 2.599180000 -6.940076000 2.497099000

H 0.878088000 -7.314760000 2.218538000

C -5.174220000 -4.372869000 2.426247000

H -5.793150000 -4.486596000 1.520934000

H -4.814775000 -5.369676000 2.726140000

H -5.839419000 -3.995608000 3.220503000

C -1.366921000 7.391407000 1.547191000

H -2.137959000 7.656135000 2.289858000

H -0.484874000 8.023277000 1.734422000

H -1.770570000 7.666309000 0.556782000

C 5.520617000 5.208671000 1.657139000

H 5.258564000 6.149530000 2.166821000

H 6.459923000 4.833041000 2.092534000

H 5.727060000 5.460269000 0.600820000

C -0.220282000 -7.323395000 -1.389107000

H 0.598241000 -7.955492000 -1.767732000

H -0.398681000 -7.613887000 -0.338294000

H -1.133580000 -7.569356000 -1.953712000

**1M-H-:** E= -4110.707534

C 4.210671000 4.519729000 1.233278000

H 3.965976000 5.576037000 1.124971000

C 3.515319000 2.195113000 1.486435000

C 1.181214000 -1.225049000 -1.761815000

C -0.918728000 0.689086000 -1.711308000

C 3.182470000 3.579434000 1.408774000

C 2.252612000 -2.195878000 -1.766584000

C 5.553394000 4.149170000 1.173963000

C -0.602891000 3.400241000 1.547042000

C 1.110313000 1.593508000 1.605320000

C 2.475234000 1.196387000 1.580619000

C -1.984143000 1.656009000 -1.717652000

C -1.234661000 -0.700309000 -1.660091000

C 0.084151000 0.610480000 1.695931000

C 4.523995000 2.392815000 -1.914841000

H 4.778631000 3.451472000 -1.917594000

C 1.962140000 -3.593534000 -1.730448000

C 4.551196000 -1.972342000 1.580323000

C 5.236669000 0.387949000 1.504492000

C 3.613742000 -1.774375000 -1.795325000

C 5.871334000 2.797285000 1.305013000

H 6.923199000 2.517728000 1.261436000

C 0.436706000 1.120753000 -1.794580000

C 2.814443000 -0.185758000 1.618614000

C 1.488140000 0.165149000 -1.805454000

C 0.763544000 2.996036000 1.546794000

C 3.023103000 -4.510006000 -1.723742000

H 2.816066000 -5.579055000 -1.691726000

C 4.201700000 -0.588906000 1.575559000

C -0.174921000 -1.658925000 -1.704892000

C 3.176817000 1.995679000 -1.895911000

C 2.863317000 0.605421000 -1.853911000

C 1.787669000 -1.168975000 1.681096000

C -1.301587000 1.012994000 1.711266000

C 0.421949000 -0.771796000 1.734739000

C 5.567929000 1.471736000 -1.892918000

C -0.623530000 -1.769193000 1.800633000

C 4.887070000 1.807949000 1.444575000

C -2.609820000 -1.130971000 -1.495440000

C -8.418252000 -3.446041000 0.585506000

H -8.982221000 -4.368473000 0.750607000

C 5.904617000 -2.332983000 1.510549000

H 6.187012000 -3.385436000 1.490951000

C 0.569876000 -4.040878000 -1.685670000

C -2.338434000 0.042068000 1.810914000

C 0.739069000 2.531196000 -1.858852000

C 4.632427000 -2.741280000 -1.769343000

H 5.676204000 -2.430939000 -1.760807000

C -1.654069000 2.385385000 1.575974000

C -1.706194000 3.045545000 -1.852150000

C -0.299683000 -3.157269000 1.767852000

C -0.474526000 -3.073351000 -1.670603000

C -0.313658000 3.494527000 -1.876481000

C -2.899596000 -2.535355000 -1.471624000

C -5.188204000 -2.027065000 -0.848883000

C -6.306091000 1.390824000 0.360902000

C -3.645623000 -0.165578000 -1.297861000

C -0.910906000 4.768175000 1.519182000

H -1.952972000 5.086919000 1.529054000

C -6.490793000 -2.284440000 -0.293265000

C -1.076623000 -5.846996000 -1.598268000

C -1.833476000 -3.511195000 -1.600598000

C -8.230786000 -1.022199000 0.866569000

H -8.634322000 -0.092616000 1.273108000

C -3.000075000 2.728120000 1.405800000

H -3.261944000 3.748148000 1.135224000

C 3.493458000 -2.984050000 1.624976000

C 2.092879000 2.979026000 -1.901594000

C -1.338051000 -4.099427000 1.777006000

H -1.110533000 -5.162618000 1.709102000

C 3.927358000 -0.345058000 -1.841570000

C 5.252005000 0.113175000 -1.861015000

H 6.075554000 -0.598724000 -1.823070000

C 4.358184000 -4.105541000 -1.731914000

C -3.341653000 1.221225000 -1.595838000

C -3.675952000 0.457727000 1.758416000

H -4.477575000 -0.266224000 1.857190000

C -1.992075000 -1.377931000 1.861509000

C -4.220417000 -2.937797000 -1.221831000

H -4.482895000 -3.994867000 -1.202560000

C 1.780215000 3.990640000 1.480212000

C 6.926596000 -1.384044000 1.449984000

C -7.194388000 -3.478931000 -0.081618000

H -6.781009000 -4.429167000 -0.434454000

C 0.246538000 -5.408405000 -1.634716000

H 1.036004000 -6.158828000 -1.621887000

C -7.003104000 -1.024961000 0.160468000

C 0.078472000 5.750739000 1.481095000

C 1.412127000 5.345560000 1.469826000

H 2.180691000 6.117692000 1.446201000

C 2.129282000 -2.574149000 1.674396000

C 6.575221000 -0.035516000 1.454685000

H 7.377259000 0.700078000 1.405611000

C -6.044884000 0.023822000 -0.139639000

C 3.793554000 -4.353861000 1.595778000

H 4.830910000 -4.684053000 1.556489000

C -4.364824000 2.159996000 -1.807561000

H -5.393626000 1.811823000 -1.840793000

C -7.572767000 1.951801000 0.060255000

H -8.237272000 1.378752000 -0.589345000

C 0.015588000 4.859551000 -1.899934000

H -0.777421000 5.606493000 -1.880308000

C -4.026962000 1.778889000 1.469677000

C -2.777218000 3.951928000 -1.951201000

H -2.582489000 5.019591000 -2.053185000

C 2.356649000 4.356183000 -1.935930000

H 3.385851000 4.713300000 -1.948555000

C 1.335648000 5.306234000 -1.924963000

C -8.921806000 -2.215223000 1.062154000

H -9.872210000 -2.199245000 1.605294000

C -2.094386000 -4.894580000 -1.596439000

H -3.124510000 -5.249546000 -1.572546000

C -4.926368000 -0.600362000 -0.790707000

C -4.105060000 3.520153000 -1.972081000

C -5.423390000 2.184537000 1.157389000

C 1.104874000 -3.565837000 1.698294000

C -2.987765000 -2.366996000 1.881813000

H -4.038529000 -2.084300000 1.880247000

C -7.984885000 3.196586000 0.526328000

H -8.972179000 3.581073000 0.253978000

C 2.798708000 -5.330508000 1.583151000

C -2.681603000 -3.724558000 1.827311000

C 1.467972000 -4.919662000 1.640189000

H 0.695144000 -5.686470000 1.618832000

C -7.125385000 3.950391000 1.332423000

H -7.431159000 4.924298000 1.724095000

C -5.863578000 3.439387000 1.626848000

H -5.192720000 4.019648000 2.264912000

C 3.148216000 -6.790214000 1.433903000

H 3.133299000 -7.082825000 0.368399000

H 4.155932000 -7.011851000 1.819185000

H 2.429092000 -7.439083000 1.958061000

C -3.770873000 -4.766251000 1.772981000

H -4.762122000 -4.309800000 1.635779000

H -3.597493000 -5.457366000 0.931758000

H -3.794766000 -5.373249000 2.694855000

C 8.369635000 -1.822862000 1.380391000

H 8.529100000 -2.540366000 0.557952000

H 9.048281000 -0.971044000 1.220961000

H 8.680048000 -2.328693000 2.311048000

C 6.636500000 5.169415000 0.920240000

H 7.545636000 4.949269000 1.502661000

H 6.929661000 5.173783000 -0.145116000

H 6.306731000 6.188610000 1.174216000

C 7.009975000 1.914023000 -1.872867000

H 7.541791000 1.469094000 -1.015475000

H 7.543659000 1.594339000 -2.784607000

H 7.099457000 3.007948000 -1.793724000

C 1.661489000 6.778370000 -1.880413000

H 1.898266000 7.092367000 -0.848569000

H 2.537393000 7.020608000 -2.503645000

H 0.816179000 7.394705000 -2.224025000

C -5.251942000 4.490752000 -2.111106000

H -5.817958000 4.555231000 -1.166350000

H -4.905390000 5.502116000 -2.375683000

H -5.964154000 4.159067000 -2.884493000

C -1.411406000 -7.317736000 -1.518334000

H -2.214956000 -7.587179000 -2.223423000

H -0.537592000 -7.949645000 -1.741263000

H -1.768694000 -7.588745000 -0.508695000

C 5.464199000 -5.128263000 -1.648464000

H 5.334595000 -5.929100000 -2.395419000

H 6.454337000 -4.673359000 -1.805375000

H 5.473228000 -5.613865000 -0.656725000

C -0.290975000 7.210240000 1.385644000

H 0.510353000 7.861209000 1.769116000

H -0.468495000 7.495965000 0.333357000

H -1.213453000 7.434907000 1.943891000

# **VII. References**

[1] a) N. V. Kozhemyakina, J. Nuss, M. Jansen, *Z.* *Anorg. Allg. Chem.* **2009**, *635*, 1355–1361. b) W. Stawski, Y. Zhu, Z. Wei, M. A. Petrukhina, H. L. Anderson, *Chem. Sci.* **2023**, *14* (48), 14109–14114.

[2] J. Lión-Villar, J. M. Fernández-García, S. Medina Rivero, J. Perles, S. Wu, D. Aranda, J. Wu, S. Seki, J. Casado, N. Martín, *Nat. Chem.* **2025**, DOI: 10.1038/s41557-025-01810-2

[3] Rigaku Corp. Rigaku Oxford Diffraction, CrysAlisPro Software System, Version 1.171.43.92a, **2023**.

[4] Rigaku Oxford Diffraction. SCALE3 ABSPACK: A Rigaku Oxford Diffraction Program (1.0.11,Gui:1.0.7) (C) **2005**.

[5] G. M. Sheldrick, *Acta Crystallogr.* **2015**, *A71*, 3–8.

[6] G. M. Sheldrick, *Acta Crystallogr.* **2015**, *C71*, 3–8.

[7] O. V. Dolomanov, L. J. Bourhis, R. J. Gildea, J. A. K. Howard, H. Puschmann, *J. Appl. Crystallogr.* **2009**, *42*, 339–341.

[8] Gaussian 09, Revision D.01, M. J. Frisch, G. W. Trucks, H. B. Schlegel, G. E. Scuseria, M. A. Robb, J. R. Cheeseman, G. Scalmani, V. Barone, G. A. Petersson, H. Nakatsuji, X. Li, M. Caricato, A. Marenich, J. Bloino, B. G. Janesko, R. Gomperts, B. Mennucci, H. P. Hratchian, J. V. Ortiz, A. F. Izmaylov, J. L. Sonnenberg, D. Williams-Young, F. Ding, F. Lipparini, F. Egidi, J. Goings, B. Peng, A. Petrone, T. Henderson, D. Ranasinghe, V. G. Zakrzewski, J. Gao, N. Rega, G. Zheng, W. Liang, M. Hada, M. Ehara, K. Toyota, R. Fukuda, J. Hasegawa, M. Ishida, T. Nakajima, Y. Honda, O. Kitao, H. Nakai, T. Vreven, K. Throssell, J. A. Montgomery, Jr., J. E. Peralta, F. Ogliaro, M. Bearpark, J. J. Heyd, E. Brothers, K. N. Kudin, V. N. Staroverov, T. Keith, R. Kobayashi, J. Normand, K. Raghavachari, A. Rendell, J. C. Burant, S. S. Iyengar, J. Tomasi, M. Cossi, J. M. Millam, M. Klene, C. Adamo, R. Cammi, J. W. Ochterski, R. L. Martin, K. Morokuma, O. Farkas, J. B. Foresman, and D. J. Fox, Gaussian, Inc., Wallingford CT, 2016.

[9] a) A. D. Becke, *J. Chem. Phys*. **1993**, *98*, 5648–5652. b) C. Lee, W. Yang, R. G. Parr, *Phys. Rev. B* **1998**, *37*, 785–789. c) S. H. Vosko, L. Wilk, M. Nusair, *Can. J. Phys*. **1980**, *58*, 1200–1211.

[10] S. Grimme, J. Antony, S. Ehrlich, H. Krieg, H. *J. Chem. Phys*. **2010**, *132*, 154104.

[11] F. Weigend, R. Alhrichs, *Phys. Chem. Chem. Phys*. **2005**, *7*, 3297–3305.

[12] J. W. McIver, A. K. Komornicki, *J. Am. Chem. Soc.* **1972**, *94*, 2625–2633.

[13] Z. Zhou, J. M. Fernández-García, Y. Zhu, P. J. Evans, R. Rodríguez, J. Crassous, Z. Wei, I. Fernández, M. A. Petrukina, N. Martin, *Angew. Chem. Int. Ed*. **2022**, *61*, e202115747.

[14] Z. Chen, C. S. Wannere, C. Corminboeuf, R. Puchta, P. v. R. Schleyer, *Chem. Rev.* **2005**, *105*, 3842–3888.

[15] K. Wolinski, J. F. Hinton, P. Pulay, *J. Am. Chem. Soc*. **1990**, *112*, 8251–8260.
